# Overlooked poor-quality patient samples in sequencing data impair reproducibility of published clinically relevant datasets

**DOI:** 10.1186/s13059-024-03331-6

**Published:** 2024-08-16

**Authors:** Maximilian Sprang, Jannik Möllmann, Miguel A. Andrade-Navarro, Jean-Fred Fontaine

**Affiliations:** 1 Faculty of Biology, Johannes Gutenberg-Universität Mainz, Biozentrum I, Hans-Dieter-Hüsch-Weg 15, Mainz, 55128 Germany; 2 Central Institute for Decision Support Systems in Crop Protection (ZEPP), Rüdesheimer Str. 60-68, Bad Kreuznach, 55545 Germany

**Keywords:** Quality, RNA-seq, ChIP-seq, Quality markers, Clinical datasets, Bioinformatics

## Abstract

**Background:**

Reproducibility is a major concern in biomedical studies, and existing publication guidelines do not solve the problem. Batch effects and quality imbalances between groups of biological samples are major factors hampering reproducibility. Yet, the latter is rarely considered in the scientific literature.

**Results:**

Our analysis uses 40 clinically relevant RNA-seq datasets to quantify the impact of quality imbalance between groups of samples on the reproducibility of gene expression studies. High-quality imbalance is frequent (14 datasets; 35%), and hundreds of quality markers are present in more than 50% of the datasets. Enrichment analysis suggests common stress-driven effects among the low-quality samples and highlights a complementary role of transcription factors and miRNAs to regulate stress response. Preliminary ChIP-seq results show similar trends. Quality imbalance has an impact on the number of differential genes derived by comparing control to disease samples (the higher the imbalance, the higher the number of genes), on the proportion of quality markers in top differential genes (the higher the imbalance, the higher the proportion; up to 22%) and on the proportion of known disease genes in top differential genes (the higher the imbalance, the lower the proportion). We show that removing outliers based on their quality score improves the resulting downstream analysis.

**Conclusions:**

Thanks to a stringent selection of well-designed datasets, we demonstrate that quality imbalance between groups of samples can significantly reduce the relevance of differential genes, consequently reducing reproducibility between studies. Appropriate experimental design and analysis methods can substantially reduce the problem.

**Supplementary Information:**

The online version contains supplementary material available at 10.1186/s13059-024-03331-6.

## Background

Lack of reproducibility is a major concern in biomedical research, for example in clinical studies, neuroscience, or cancer biology [1–3], and also in other scientific fields such as artificial intelligence, drug discovery, or computer science [4–6]. There have been already many publications and initiatives to address the problem such as community and statistically driven guidelines for publication or data deposition in scientific repositories [7–12]. In functional genomics, complex sequencing technologies were instrumental in producing an unprecedented amount of data covering a great variety of topics relevant to life sciences. Community-derived experimental guidelines [13] and the latest computational and mathematical methods to produce and analyze results from those technologies did not solve the reproducibility problem. Gene expression studies based on RNA sequencing are a prominent example where reproducibility is limited by factors such as batch effect or quality differences between groups of biological samples. Although methods have been proposed to identify and correct batch effects [14–16], the impact of quality imbalance (QI) between groups of biological samples or patients is largely ignored in the biomedical literature. It is therefore critical to characterize the impact of quality differences on gene expression to enable studies that can be successfully reproduced.

Gene-expression analysis of clinical datasets is impacted by various factors such as sample extraction conditions [17], experimental protocols [18], batch effects, or non-homogeneous sample quality [19]. Methods correcting batch effect from the data have been used successfully to integrate data from different batches or from independent datasets although they should be used only when necessary, as they could remove biological signals from the data, and do not address the problem of factors highly confounded with study design (sample groups being compared) [16, 20]. Unfortunately, design-quality problems are rarely considered in published studies and are often difficult or impossible to derive from open-access datasets commonly used for testing reproducibility [19]. Very few studies report comprehensive quality control results necessary to evaluate the quality of the samples [21]. Quality metrics are also not perfect and their usefulness can be highly specific to the experimental conditions including cell and assay types [12]. The poor reporting or documentation of methods, data, and analysis results is also a problem for reproducibility [22]. Although it is a common practice to filter out mitochondrial and ribosomal genes from gene-expression data [23, 24], this is not always possible or desired, for example when studying topics related to mitochondria, respiration, or programmed cell death. In addition, there could be other genes equally related to sample quality that should be considered. In statistics, the impact of a confounding factor not considered, such as the sample quality, is known as the omitted variable bias [25]. Depending on the correlation of this factor to the dependent or independent variables, it can lead to hide, reverse, strengthen, or weaken an effect under study (e.g., the expression of a gene as a result of a variable condition). Taken together, given the complexity of biomedical experiments and the low reporting standard of the literature, the impact of variable sample quality on biomedical results and thus on reproducibility still needs to be characterized.

In order to increase our understanding about why reproducibility is low in clinically relevant RNA-seq results, we have studied 40 disease-related datasets. The public availability of many published datasets together with recent quality-related research in machine learning gives the opportunity to discover factors of reproducibility at the level of gene expression [26]. We studied the impact of quality imbalance on the number of differential genes associated with groups of disease and control patients in each dataset. We also searched if some genes could correlate with sample quality in the datasets. Finally, we evaluated the reproducibility potential of differential gene lists derived from different datasets depending on quality imbalance and showed preliminary results on ChIP-seq data.

## Results

In order to better understand the impact of low-quality samples in RNA-seq datasets on reproducibility, we have stringently selected 40 publicly available and clinically relevant human datasets comparing disease to control patient samples (Additional file 2: Table S1). The stringency of this selection aimed at minimizing potential confounding factors that would prevent us from observing the impact of sample quality over gene expression. Accordingly, we chose groups of patients that were as homogeneous as possible within each dataset based on provided clinical information such as age range, gender ratio, and other clinical features when available. We then derived the quality of each sample as a probability of being of low quality from an accurate machine learning algorithm [26]. Important to our study was the definition of a quality imbalance (QI) index for each dataset which ranges from 0 to 1, where 0 means that quality is not confounded with the groups, and 1 means that quality is fully confounded with the groups (see the “Methods” section for details).

### Dataset quality

**Fig. 1:**
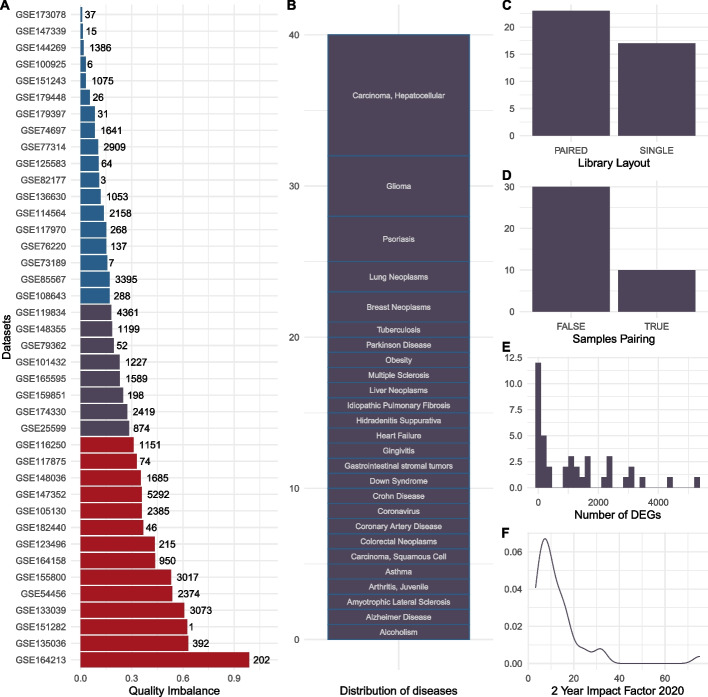
Quality imbalance of the 40 datasets. Clinically relevant human datasets were selected for patient samples’ homogeneity in the comparison groups (control vs disease groups). **A** quality imbalance (QI) index (*x*-axis) of each dataset (*y*-axis). The QI index is calculated as the absolute correlation coefficient between the samples’ probabilities of being of low quality and their groups. If its QI index is above 0.3, a dataset is considered highly imbalanced (red bars). If it is less than 0.18, the QI index is considered low (blue bars). The number of significant differential genes is given as annotation to the bars. **B** stacked barplot for the number of samples (*y*-axis) in each dataset. **C** number of datasets designed with sample pairing. **D** number of datasets sequenced with either single- or paired-end reads. **E** distribution of journal impact factors for the published articles related to the datasets

From the 40 datasets, our stringent manual selection included a total of 1164 human samples. There was an average number of samples equal to 29.1 per dataset ranging from 8 to 96. Our selection defined two equally sized groups of samples per dataset as disease and control. Fourteen (35%) datasets had a high QI index above 0.30 (Fig. 1A). Note that various statistical methods or tests provide comparable numbers of high-QI datasets, ranging from 9 to 15 (Additional file 1). In total, twenty-six diseases were covered (Fig. 1B). Eleven (27.5%) datasets had few significant differential genes (*n* ≤ 50) (Fig. 1E). The QI index moderately correlated positively with pair-ended library layout (*r* = 0.266), moderately correlated negatively with 5-year journal impact factors (*r* =  − 0.272), but did not correlate with paired dataset design (*r* = 0.03). Figure 1C to F gives an overview of the library layout of the datasets, as well as whether datasets had paired control and disease samples, the number of differentially expressed genes, and the impact factors of the journals in which the data were published. We did a similar analysis for ChIP-seq data sets and found that 3 out of 10 datasets had a high QI index, indicating that such quality imbalance exists in other assays too (Additional file 3: Table S2, Additional file 1: Fig. S1; see the “Methods” section for details).

### Impact of quality imbalance on differential gene analysis results

#### Analysis of data subsets of the same size

**Fig. 2:**
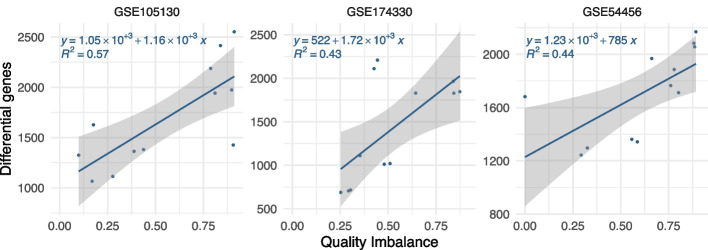
QI and differential genes in data subsets. For three large datasets (panels) in our study, we randomly sampled several smaller subsets (points) of 20 samples each to compare their number of differential genes to their respective QI indices in equally sized and sourced datasets (see the “Methods” section for details). This simulation allows us to isolate and observe the effect of a quality imbalance on the number of differential genes from the effect of any other confounding factors such as particular patient characteristics (e.g., age, gender, or ethnic group) or dataset size (number of samples). For each dataset, we can observe a positive correlation between the quality imbalance and the number of differential genes of its subsets. Gray areas indicate confidence intervals

Working with three of the largest datasets (GSE105130, GSE174330, and GSE54456) and the quality probabilities associated with their samples, we built several data subsets based on different selections of patients in the control and disease groups to represent low- and high-QIs. In a differential gene analysis, the larger the patient groups, the more powerful the statistical tests, and thus the more significant differential genes can be found. Therefore, we set the same number of samples (*n* = 20) to each data subset to focus our observations on the impact of QI on the number of differential genes derived by comparing the disease and control groups in each subset. The analysis of the subsets of the three selected datasets shows a clear linear relationship between QI and the number of differential genes (*R*^2^ = 0.57, 0.43, and 0.44, respectively): the higher the QI, the more the differential genes (Fig. 2). For those 3 datasets, an increase of the QI from 0 to 1 translates into an increase of 1222 differential genes on average (1160, 1720, and 785, respectively). This large variability might be due to a synergistic effect between quality and other confounding factors in some subsets that could have been created by the random selection of a limited number of patients per group, although patient characteristics are comparable between groups when considering full datasets.

#### Analysis of 40 full datasets of various sizes

**Fig. 3:**
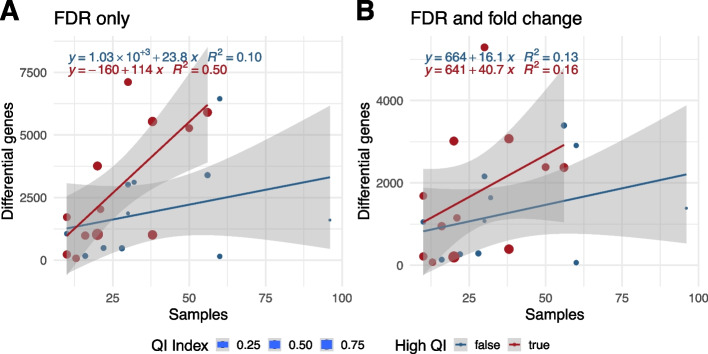
Impact of quality imbalance (QI) on the number of differential genes. On the scatter plots, points represent datasets colored by quality imbalance status: low (blue; QI index ≤ 0.3) or high (red; QI index > 0.3). The plotted datasets have each a minimum of 50 significant differential genes. *X*-axis indicates the number of samples and *y*-axis the number of differential genes in the datasets. Solid blue and red lines show linear regression results (confidence interval in gray). On panel **A**, the number of differential genes was derived by using a false discovery rate (FDR) cutoff in the differential analysis, while on panel **B** this number was derived by using both an FDR cutoff and a fold change cutoff. Panel **A** shows a faster increase of the number of differential genes in relation to the number of samples for high QI datasets, while on panel **B** this difference is reduced

The impact of the QI could also be observed on the 40 full datasets with different characteristics including other diseases and various numbers of samples (Fig. 3 and Additional file 1: Fig. S2). Based on linear regressions, the number of differential genes derived using a false discovery rate cutoff (FDR < 0.05) increased four times faster with the dataset size for highly imbalanced datasets compared to more balanced datasets (slope = 114 vs 23.8, respectively; Fig. 3A). When removing datasets designed with sample pairing from the analysis, we observed a similar difference (slope = 108 vs 23.5, respectively; Additional file 1: Fig. S2A). Analyzed separately, a smaller number of paired-sample datasets showed a similar trend (Additional file 1: Fig. S2C). We could also observe that deriving differential genes using not only an FDR cutoff but also a fold-change cutoff decreased the slope for the high-QI datasets and consequently considerably reduced the differences (Fig. 3B and Additional file 1: Fig. S2B and D).

### Recurrence of quality-associated genes

In order to identify quality-associated genes occurring in several datasets (quality markers), we analyzed 13 datasets with the lowest QI indices (index ≤ 0.18; only one dataset per disease). We considered a gene to be a low- or a high-quality marker if its expression significantly correlated with the low- or high-quality of the samples, respectively.

**Fig. 4:**
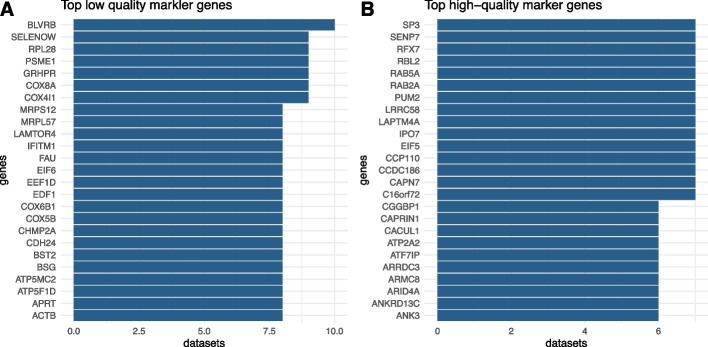
Top 25 markers of quality. **A** low-quality marker genes. **B** high-quality marker genes. Genes whose expression correlates positively or negatively with low sample quality in multiple datasets were considered low- or high-quality markers, respectively. Computations were done on 13 datasets with the lowest QI index values (QI index < 0.18) and each representing a different disease. Comprehensive gene lists, including more low-quality markers found in 8 datasets and more high-quality markers found in 6 datasets are provided as supplementary material (Additional file 4: Table S3 and Additional file 6: Table S5)

**Fig. 5:**
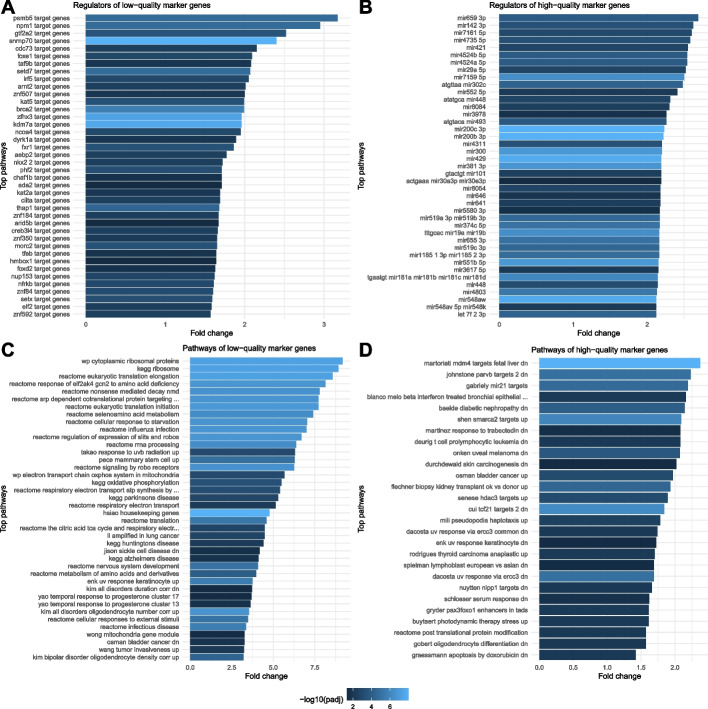
Gene set enrichment analysis. Database annotations of the low- and high-quality markers were used to find related regulators (top regulators in panels **A** and **B**, respectively) and pathways (top pathways in panels **C** and **D**, respectively). In the regulatory enrichment analysis, we found a regulation of low-quality markers by transcription factors (**A**; *n* = 50), while high-quality markers are mostly regulated by miRNAs (**B**; *n* = 302; 89% miRNAs and 11% transcription factors). In the pathway enrichment analysis, low-quality markers were notably enriched in mitochondria-related and ribosomal pathways. The pathway enrichment analysis for high-quality markers found various regulators and diseases

We found a total of 7708 low-quality markers occurring in at least 2 (15%) out of 13 datasets (Fig. 4A, Table 1 and Additional file 4: Table S3). There were low-quality markers occurring in up to 10 (77%) datasets. The list of top low-quality markers was significantly enriched in targets of 48 transcription factors which could be themselves low- (e.g., snrnp70, thap1, psmb5) or high-quality markers (e.g., setd7, fxr1) (Fig. 5A and Additional file 5: Table S4). The list was also enriched in various molecular pathways, including the expected mitochondria-related pathways (e.g., cell respiration and oxidative phosphorylation) and ribosomal pathways, but also other pathways such as response to starvation, response to ultra-violet radiation, housekeeping genes (largely overlapping mitochondrial and ribosomal pathways) and various diseases such as influenza (not included in our dataset selection), neuro-degenerative and cancer diseases (Fig. 5C).

**Table 1:** Recurrence of quality makers in selected datasets. Thirteen datasets with low-quality imbalance were used to derive quality marker genes. Genes whose expression correlated positively or negatively with low sample quality in at least 2 datasets were defined as low-quality or high-quality markers, respectively

Recurring datasets	Low-quality markers	High-quality markers
2 (15%)	7708	5243
3 (23%)	3443	2405
4 (31%)	1597	951
5 (38%)	724	287
6 (46%)	320	76
7 (54%)	136	15
8 (62%)	51	0
9 (69%)	7	0
10 (77%)	1	0

We found a total of 5243 high-quality markers occurring in at least 2 (15%) out of 13 datasets (Fig. 4B, Table 1 and Additional file 6: Table S5). There were high-quality markers occurring in up to 7 (54%) datasets. The list of top high-quality markers was significantly enriched in targets of 306 regulators including 280 (91.5%) miRNAs (Fig. 5B and Additional file 7: Table S6). There were also 19 (6.2%) transcription factors but they were not represented in the top 100 regulators of the gene set enrichment analysis. Some of these transcription factors were observed as low- (e.g., kdm7a or tfeb) or high-quality markers (e.g., taf9b or znf184). The list was also enriched in various molecular pathways, including some diseases (e.g., skin carcinogenesis, bladder cancer, uveal melanoma, diabetic nephropathy) and other pathways (e.g., uv response, serum response) (Fig. 5D).

### Recurrence of quality-associated genes in multiple data types

Adapting the approach above, we derived quality marker genes using protein-DNA binding data from 10 human ChIP-seq datasets targeting the H3K27ac histone mark. When considering only genes that arise in more than 20% of datasets in each data type (RNA-seq or ChIP-seq), we see an overlap of low- and high-quality marker genes of 438 and 298, respectively (Additional file 8: Table S7 and Additional file 10: S9). For the molecular pathways related to those markers in each data type, we see an overlap of pathways for low- and high-quality marker genes of 5 and 11, respectively (Additional file 9: Table S8 and Additional file 11: S10).

Since in ChIP-seq analysis, mitochondrial genes are blacklisted [27], they cannot be found in these overlaps. However, we still find the related pathway for oxidative phosphorylation enriched in the ChIP-seq analysis for low-quality markers, and subsequently in the overlap with RNA-seq-derived pathways.

### Impact of quality on reproducibility

As seen above, quality markers were identified in a substantial proportion of the datasets. Considering the lists of significant differentially expressed genes from those datasets, if the quality markers are also found in the lists, they will reduce the reproducibility between gene-expression studies.

**Fig. 6:**
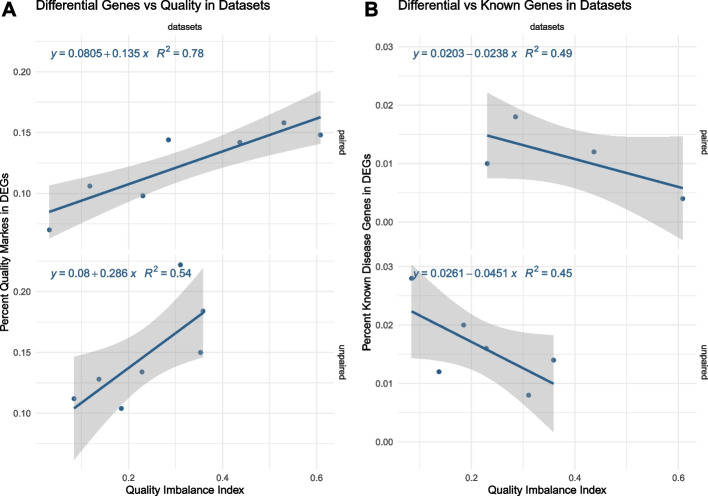
Quality and disease gene proportions in differential genes. To test the presence of quality markers or known disease genes in lists of differentially expressed genes (DEGs), we selected datasets with 50 samples or less, with at least 500 differential genes, and about a disease with at least 50 known genes. Only the top 500 DEGs per dataset and the top 50 known disease genes per disease were used in this analysis. For those datasets (black points on the plots), the proportion of potential quality markers (**A**/left) or known disease genes (**B**/right) in the DEGs on the *y*-axis is compared to the dataset QI index on the *x*-axis. Sample pairing design of the datasets is detailed (paired or unpaired design)

The proportion of quality markers in the top 500 differential genes had a strong linear relationship with the QI index of the related datasets: the higher the QI index, the higher the proportion of quality markers in the differential genes (Fig. 6A). The proportion increased approximately 2 times faster for unpaired datasets than for paired datasets This proportion was equal in average to 13% and could represent up to 22% of the differential genes (namely, 110 genes out of the top 500 differential genes).

For the same datasets, we also compared the proportion of the top 50 known disease genes in the top 500 differential genes to the QI index (Fig. 6B). There was a negative linear relationship: the higher the QI index, the lower the proportion of known disease genes in the differential genes. The proportion decreased approximately 2 times faster for unpaired datasets than for paired datasets. In 10 selected diseases (with more than 300 associated disease genes), potential markers for low- and high-quality constitute up to 19% and 9% of the 300 top-associated disease genes, respectively (Additional file 12: Table S11). This proportion was significant or marginally significant for high-quality markers in half of the diseases (e.g., Alzheimer’s disease, colorectal neoplasms, amyotrophic lateral sclerosis) and significant for the low-quality markers in two datasets: Parkinson’s disease and amyotrophic lateral sclerosis. Notably, based on the literature analysis, quality markers cannot be easily filtered out from lists of disease genes as they could be highly ranked as known disease genes (Additional file 1: Fig. S3).

### Quality imbalance mitigation

The low- or high-quality information of the samples provided by machine learning was used either as a confounding factor in the differential gene analysis or to remove quality outlier samples from the datasets before the analysis, to see if it could impact the results of the downstream pathways enrichment analysis (Additional file 13: Table S12).

When using the quality information as a confounding factor in the differential gene analysis, we could observe a reduction of low-quality marker pathways in the results of the downstream gene set enrichment analysis. However, the results did not change for most datasets. When using the quality information to filter out outlier samples, we observed a strong decrease in low-quality marker pathways for almost all datasets. A combination of both methods did not further impact the outcome of the enrichment analysis. 

## Discussion

While Next Generation Sequencing has revolutionized biomedical science with an unprecedented amount of data and novel clinically relevant applications, reproducibility between research results is still limited even in well-designed studies. Although reasons for low reproducibility in poorly designed studies may be easily identified (e.g., imbalanced sex ratio or large age difference between sample groups), they are not obvious for well-designed studies. We hypothesized that differences between the quality of the samples within a study may bias the results and negatively impact reproducibility. Therefore, we studied 40 well-designed and clinically relevant datasets in order to evaluate and quantify the impact of sample quality differences on significant differential genes (disease vs control samples). We found that the expression profile of many genes correlated with quality, we classified those genes as markers of quality, and we were able to relate them to molecular regulators. Finally, we quantified the negative impact of QI between sample groups on the relevance of top differential genes.

Although the GEO database has only minimal requirements for standardized metadata [28], it is still the largest repository for gene expression datasets and contains a great variety of clinically relevant datasets. By exploring the GEO database to find RNA-seq datasets, we have seen a large majority of poorly designed datasets and only a minority of datasets that could meet our inclusion standards. Patient sample metadata was often inconsistently distributed across the three data and information sources considered (scientific journal article, GEO, and SRA databases) and in different locations within each source (main text and supplementary files in journals, web pages, and downloaded metadata files in GEO and SRA). Notably, the GEO and SRA web pages often contained considerably less information than the corresponding downloaded metadata files (GEOSeries and SraRunTable files, respectively). Yet, the metadata was, in general, not sufficient to identify common confounding factors such as age and gender. Samples metadata could also be provided as summary tables, including age and gender balance in percentages within each comparison group but with no possibility to trace the information back at the sample level [29–31]. As another example, batch effect or sample pairing could be recognized in the journal article, but related information could not be found or was unclear to identify the corresponding samples [32–37]. Nevertheless, we used the available information to include 40 well-designed datasets according to our criteria. The latter were also met thanks to a sub-selection of samples in several datasets to ensure homogeneity between the comparison groups (controls vs patient samples with similar age ranges and gender ratios). Because good experimental designs minimize the effect of common confounders (including batch) [38], we could focus our observations on the effect of sample quality on gene expression.

Sample quality was rarely mentioned in the dataset articles or databases and specific sample-level quality information was provided only for a very few datasets using the RNA integrity number [39, 40]. Definitively useful to filter out samples of the lowest quality, the RNA integrity number is not commonly used for a fine classification by quality [41]. This lack of reporting may indicate the low importance experimentalists place on further quality-related analyses after data generation. Thanks to a finer classification of sample quality using machine learning models, we observed that 35% of well-designed gene-expression datasets had a high QI between comparison groups (QI index > 0.3). Importantly, this result derived from 40 independent, well-designed datasets free from other confounding factors proves that a substantial proportion of the published datasets in the biomedical literature is flawed by quality problems. A comparable percentage was found in ChIP-seq experiments. It should be noted that when running the analysis with more robust and non-parametric tests like Spearman’s correlation and the median-based central tendency difference [42], this ratio stayed rather stable at around 30%. Unfortunately, we found many more poorly designed or poorly described datasets in the repositories for which additional confounding factors such as imbalances in age, gender, weight, or active medication should be considered and which we could not include, since they were not stratifiable. Interestingly, there was only a moderate negative correlation between QI index and journal impact factor.

The impact of those quality differences between sample groups has been mainly overlooked in the literature (Fig. 1). The number of significant differential genes is expected to increase with the number of samples (definition of statistical tests), and it has been shown that this relation could be linear in gene expression studies [43]. Irrespective of the number of samples, we found that QI has a direct impact on the number of differential genes: the higher the QI, the more the differential genes (Figs. 2 and 3). Between a perfectly balanced dataset and a perfectly imbalanced dataset, the number of differential genes could increase by 1222 genes on average. Selecting differential genes using both FDR and fold change cutoffs as done by default in our study seems to considerably reduce this difference in comparison to using FDR cutoffs only (Fig. 3), confirming a common usage in the literature. These results partly explain the difficulty of studies based on current sequencing technologies to derive relevant small gene-expression changes.

Thanks to the comparison of gene expression profiles to sample quality across a sub-selection of the less quality-imbalanced datasets, we found many markers for low or high sample quality that can neither be associated with those diseases nor with other common confounding factors (Fig. 4). A number of markers could be identified in a large proportion of the studied datasets (up to 77%). We found enrichment of (i) targets of transcription factors (e.g., psmb5, gtf2a2) (Fig. 5A) and (ii) mitochondria-related and ribosomal pathways [23, 44] among low-quality markers (Fig. 5C). This is consistent with the activation of additional regions of the gene regulatory network in response to stress in low-quality samples [45, 46] and confirms that those marker genes are intrinsically linked to quality. Interestingly, the enrichment among high-quality markers (genes with lower expression in low-quality samples) indicates enrichment of miRNA targets (e.g., mir659, mir142) and targets of various regulators (Fig. 5B and D, respectively). This result could reflect the regulatory role of miRNAs activated in the low-quality samples in regulating stress response, cell repair, or cell proliferation mechanisms [47–51], resulting in decreased gene expression of particular genes including various transcription factors in the low-quality samples. Our results across datasets and diseases identify molecular regulators (transcription factors and miRNAs) that are the most sensitive to quality changes. Research fields such as System Biology could use this information to calibrate regulatory network models which are often sensitive to small changes in their parameters [52].

Given the high proportion of datasets in the literature that are highly quality imbalanced, it is likely that many differential genes are associated with quality rather than disease. Indeed, on the one hand, we found that the proportion of quality markers in the differential gene lists (up to 22%) positively correlated with the QI index of the dataset. Many of those recurring genes could also be found to be related to QI in ChIP-seq. For example, the ChIP-seq low-quality markers were still enriched for the oxidative phosphorylation pathway, despite mitochondrial genes being removed from the analysis through blacklists. This suggests, in this context, that genes that are related to the mitochondrial environment or mitochondrial pathways hold similar information about quality status as the mitochondrial RNA transcripts themselves. On the other hand, the proportion of known disease genes negatively correlated with the QI index (Fig. 6). Interestingly, those correlations were lower for paired-sample datasets. Provided that pairs of samples are not confounded with quality differences or batch processing, designing large datasets with sample pairing and applying appropriate statistics (paired statistical tests) would be the optimal way to maximize the relevance and reproducibility of RNA-seq results. We also found that known disease genes derived from the literature could be significantly enriched in quality markers (up to 19%). Although it questions the validity of the literature, we must note that RNA integrity could be impacted by a disease state, such as in some types of cancer [53]. Therefore, some genes could logically be both quality and disease markers. However, our analyses highlight the possibility that, even if recurrently observed in different experiments in the literature, some genes deemed as disease markers may actually indicate quality bias. Further investigations paired with wet-lab experiments would be required to identify those genes more precisely.

Taken together, we have demonstrated that QI between sample groups impairs the reproducibility of clinically relevant RNA-seq results. In the future, it would be interesting to reproduce our analyses with many more datasets to test statistical associations with additional clinical parameters. We already mentioned an association with tumor stage [53] but other parameters may be relevant for other diseases. The collection of studied datasets could also contain a more comprehensive selection of diseases or also non-disease datasets. It included many cancer-like diseases that could have influenced some of our results. We tried to minimize such an impact by carefully selecting the datasets involved based on the analysis. For example, quality markers were derived on a subset of the datasets with the lowest QI indices and each representing a different disease. It would be also interesting to study other sequencing data types such as ATAC-seq or ChIP-seq in more detail. Our preliminary results show the same proportion of high QI in clinically relevant human ChIP-seq datasets. Those preliminary results should be further investigated and compared to the literature about non-biologically relevant binding sites [27, 54–56]. We could also show that removing quality outliers will decrease the number of pathways of low-quality marker genes in almost all cases. We suggest using the seqQscorer tool as not only a quality control tool, but an additional possibility to search for outliers in the data, as these will not always coincide with outliers in PCA-projection [57].

## Conclusions

In conclusion, a difference of quality between sample groups of clinically relevant gene-expression datasets impairs reproducibility across studies thanks to a compendium of genes masking the relevance of true disease genes in statistical results. A large proportion of the published datasets present a substantial imbalance between the quality of the sample groups, their results are thus put into question. From individual studies showing sample quality as a major confounder in a limited number of experimental conditions [57–59], the field of genome-wide data analysis would benefit from studies accurately comparing many possible confounders and their impact on gene expression and reproducibility, although the latter is expected to be low for some diseases [59] and some quality markers would likely be specific to experimental conditions [12]. In this way, we will be able to optimize study design, moving away from generic blacklists to more rational filters of gene-based results, specific to data type and study.

## Methods

All the statistical methods, scripts and software used in this study are described in this section.

### RNA-seq datasets

Dataset metadata was derived by gathering information of the following three sources: the metadata from the GEO database (Web pages and GEO Series file), the metadata from the SRA database which hosts the raw data files (SraRunTable.csv files), and the corresponding published article (PubMed Central or publisher’s web site: main article and supplementary files if available). Starting from the GEO database, we searched those metadata sources to get 40 well-designed, publicly available, and clinically relevant RNA-seq datasets with the following criteria:

1. Assay: either single-ended or pair-ended Illumina sequencing technology.
2. Samples: human primary cells or tissues from a disease and its control group. Duplicate samples were not used.
3. Design: At least four samples per group. All samples from a same batch if documented. Paired or unpaired samples.
4. Sample groups: Samples were selected to balance the age range and gender ratio per group. If possible, only samples of the same gender were selected (either male or female). If documented, other clinical factors were used to balance the sample groups such as the BMI index, ethnicity, or disease-relevant mutations (no mutations if possible).

If samples were paired and if pairing identifiers were available at the sample level, we used the information to run appropriate analysis methods.

### Quality imbalance (QI) index and quality marker genes

For RNA-seq or ChIP-seq data, sample quality was evaluated by the seqQscorer machine learning tool [26], which returns a probability of low quality per sample: *P*_low_. For each dataset, we first derived a *P*_low_ value for each sample. Additional file 14: Table S13 contains the list of all samples and their quality features and low-quality probabilities *P*_low_ as used and returned by seqQscorer, respectively. Additional file 1: Fig. S7A shows a heatmap of FastQC’s ordinal output (0 = fail, 1 = warn, 2 = pass) and the mapping metrics of Bowtie2 in percent. Additional file 1: Fig. S7B and S7C show the distribution of uniquely mapped reads and the sequence duplication levels over bins of P_low_, two of the most relevant features for the classification of the samples by quality. Using these low-quality probabilities P_low_, we derived a quality imbalance (QI) index for each dataset to quantify how much the sample quality is confounded with the sample group (control or disease). The QI index is equal to the absolute value of Pearson’s correlation coefficient between *P*_low_ values and the sample group numerical codes (0 for control and 1 for disease, or the other way around); this is equivalent to a point-biserial correlation usually used for correlations of numerical and dichotomous variables [60]. The QI index will be equal to 0 if the quality is not confounded with the group, and it will be equal to 1 if the quality is fully confounded with the group (e.g., all disease samples have lower quality than control samples, or vice versa). Additional file 1: Fig. S8 shows exemplary datasets and their distribution of samples over *P*_low_. After visual inspection of the sample low-quality probabilities within the datasets, a QI index greater than 0.30 was considered high, and a QI index less than 0.18 was considered low. Those cutoffs were also defined to create two groups of RNA-seq datasets not too small for analytical purposes: there were 18 low-QI datasets and 14 high-QI datasets.

With RNA-seq data, quality marker genes are genes whose expression strongly correlates with *P*_low_ values independently in multiple datasets. For quality markers, a Pearson’s correlation coefficient with an absolute value greater than 0.4 was considered strongly correlated. A gene will be a low-quality marker if its expression significantly and positively correlates with *P*_low_ in multiple datasets. A gene will be a high-quality marker if its expression significantly and negatively correlates with *P*_low_ in multiple datasets.

With ChIP-seq data, quality marker bins are bins whose enrichment values (see definition below) correlate with *P*_low_ values across samples independently in multiple datasets (within each dataset, only bins with peaks in at least 3 samples were considered for correlations). For quality marker bins, a Pearson's correlation coefficient with an absolute value greater than 0.3 was considered significant. We used a less stringent cutoff as the binning and subsequent annotation of bins to the nearest gene introduces uncertainty in the gene-to-quality correlation. A bin will be a low-quality marker bin if its peak enrichment values significantly and positively correlate with *P*_low_ in multiple datasets. A bin will be a high-quality marker bin if its peak enrichment values significantly and negatively correlate with *P*_low_ in multiple datasets.

### Alternative metrics to define the QI index

As a first alternative, the QI index of a dataset was defined by the absolute value of Spearman’s correlation coefficient between *P*_low_ values of the samples and the sample group numerical codes (0 for control and 1 for disease, or the other way around). This method had the advantage of producing a QI index also ranging from 0 to 1, thus we used the same cutoff values to define the low- and high-QI groups of datasets (0.18 and 0.3, respectively). As a second alternative, the QI index of a dataset was defined by the central tendency difference, CTDiff, as defined by Lötsch and Ultsch [42]. Shortly, the CTDiff of a dataset is equal to the absolute difference of the median *P*_low_ values of the control and the disease groups of samples divided by the expected value of absolute inner differences in the dataset. This metric provides results from 0 but it has no upper limit. After manual review, we set the cutoff values to define the low- and high-QI groups of datasets to 0.5 and 1, respectively, setting a much more stringent cut-off than with the two correlation metrics we used.

### Simulated RNA-seq subsets

To study the impact of quality imbalance in similar experimental conditions, we created different subsets of 20 samples from 3 of the largest datasets in our selection (GSE105130, GSE174330, and GSE54456). The 3 datasets were selected to have at least 50 samples each and a broad distribution of *P*_low_ values associated with their samples. Each subset created was composed of 10 samples in the control group and 10 samples in the disease group from the same dataset. The 10 samples of a given group (control or disease group) were all randomly selected either from the 15 top-quality samples (lowest *P*_low_ values) or from the 15 bottom-quality samples (highest *P*_low_ values) of the same group in the source dataset. In a first iteration, for each dataset, we sampled 2 control and 2 disease groups of 10 samples each and combined them by pairs to build 4 data subsets as follows: bottom-quality control vs bottom-quality disease samples, bottom-quality control vs top-quality disease samples, top-quality control vs bottom-quality disease samples, and top-quality control vs top-quality disease samples. We performed then a total of 3 iterations to produce 12 subsets of different QI indices for each source dataset. It was not possible to always cover the theoretical range of *P*_low_ values from 0 to 1, as the groups sometimes had an intrinsic QI bias.

### Processing and analysis of RNA-seq data

The RNA-seq data was downloaded from the SRA database (single-ended reads: 10 M reads downloaded and 1 M randomly selected for analysis; pair-ended reads: 10 M read pairs downloaded and 1 M randomly selected for analysis). Sequencing reads were mapped to the transcriptome using Salmon v1.6.0 (index with decoy; quantification parameters: –seqBias –gcBias –posBias –reduceGCMemory; transcriptome GRCh38.101). Sample quality control was performed using Picard v2.23.6 and MultiQC v1.9 [61, 62]. Data manipulation was done using Samtools v1.9, seqtk v1.3, bedtools v2.29.2 [63–65]. Differential genes were derived using deseq2 v1.22.1 [66]. Except if explicitly written, significance was defined at an adjusted *p*-value < 0.05 and the absolute value of log2 fold change > 1 (*p*-values adjustment using the Benjamini–Hochberg method). Gene set enrichment analysis was done using the function fora from the fgsea package v1.20.0 [67] and gene annotations from MsigDB v7.4 [68, 69] and GS2D [70].

### Processing and analysis of ChIP-seq data

We selected 10 human ChIP-seq datasets from the GEO database that were targeting the H3K27ac histone mark and had a design containing a healthy group and a disease group. The ChIP-seq data was aligned to the genome using bowtie2 v2.3.5 and Samtools v1.9 and significant peaks were called using MACS2 v2.2.7 (narrow peak mode; adjusted *p*-value < 0.05) [71]. Peaks were assigned to genomic bins of length 500 bp with bedtools v2.29.2 (function makewindows). Bedtools functions intersect, groupby, and coverage were used to count the number of peaks per bin and generate min, max, and mean peak enrichment values for each bin.

For the gene set enrichment analysis of the ChIP-seq data, the KEGG subset of canonical pathways included in the MsigDB database version 7.4 was used along with the human reference genome hg38 (fgsea function from R package fgsea with parameters minSize = 15, maxSize = 500 and scoreType = “pos”).

### Mitigating impact of quality imbalances

When using the quality information as confounding factor in the differential gene analysis, we used 1-*P*_low_ as a continuous covariate in DESeq2 to give the samples with a higher quality the higher covariate value. This should reflect that the low-quality marker genes we found were expressed higher in the high *P*_low_ samples and the model should weight those samples lower.

When using the quality information to filter out outlier samples of a given dataset, outlier samples were defined as samples with *P*_low_ values beyond 1.5 times the interquartile range above the third quartile or below the first quartile of *P*_low_ values within the dataset (function is_outlier() from the rstatix package [72]). The impact of those 2 mitigation methods was evaluated for each dataset on the lists of pathways from the gene set enrichment analysis for the differential genes (differential pathways) and the low-quality markers (low-quality pathways). We first calculated the percentage of low-quality pathways in the differential pathways when using a mitigation method or not. We compared then the mitigation methods only for datasets for which the pathways overlap was at least equal to 1 pathway. A mitigation method was deemed to have a positive or a negative impact if the overlap decreased or increased by at least 15%, respectively.

### Supplementary Information

Additional file 1. Supplementary information and figures. This file contains a section named “Quality Imbalance based on other metrics” and the supplementary figures (Fig. S1 to Fig. S8).

Additional file 2: Table S1. Datasets Metadata RNA-seq.

Additional file 3: Table S2. Datasets Metadata ChIP-seq.

Additional file 4: Table S3. Low-Quality Marker Genes. Column N: number of datasets out of 13 in which the gene expression positively correlated with a machine-learning derived low-quality probability. Each of the 13 datasets represented a different disease and had a low-quality imbalance between control and patient groups of samples.

Additional file 5: Table S4. Regulators of the Low-Quality Marker Genes. This table shows statistically significant molecular regulators of the top 500 low-quality marker genes derived by an enrichment analysis (transcription factors or miRNAs). P-value was derived from a Fisher’s exact test and adjusted by the Bonferroni method.

Additional file 6: Table S5. High-Quality Marker Genes. Column N: number of datasets out of 13 in which the gene expression negatively correlated with a machine-learning derived low-quality probability. Each of the 13 datasets represented a different disease and had a low-quality imbalance between control and patient groups of samples.

Additional file 7: Table S6. Regulators of the High-Quality Marker Genes. This table shows statistically significant molecular regulators of the top 500 high-quality marker genes derived by an enrichment analysis (transcription factors or miRNAs). P-value was derived from a Fisher’s exact test and adjusted by the Bonferroni method.

Additional file 8: Table S7. Overlapping Low-Quality Markers in RNA- and ChIP-seq Datasets.

Additional file 9: Table S8. Overlapping Pathways enriched in Low-Quality Markers in RNA- and ChIP-seq Datasets.

Additional file 10: Table S9. Overlapping High-Quality Markers in RNA- and ChIP-seq Datasets.

Additional file 11: Table S10. Overlapping Pathways enriched in High-Quality Markers in RNA- and ChIP-seq Datasets.

Additional file 12: Table S11. Quality Markers in Known Disease Genes. For six diseases, the top 300 known disease genes were derived from the literature and compared to lists of low- or high-quality marker genes. For each disease, the table shows the number of quality markers in the disease genes and a P-value derived from a Fisher’s exact test.

Additional file 13: Table S12. Relevance of pathways enrichment after quality imbalance mitigation. Mitigation of quality imbalance was performed either within the differential analysis (using low-quality probabilities of the samples, *P*_low_ values, as covariates in DESeq2) or by removing quality outlier samples (based on *P*_low_ values). First, pathways associated with low-quality markers (low-quality pathways) were derived for various reference gene lists of the MSigDB database (column gene_sets). For each dataset, a gene set enrichment analysis was performed on differential genes (differential pathways) with a positive or negative log-fold change (pos and neg values in column deg_sign, respectively). We summarized results only for datasets for which at least 1 low-quality pathway overlaps with differential pathways (n_datasets) and counted the number of datasets where the mitigation method had positive (column better), negative (column worse), or no substantial impact (column no_change).

Additional file 14: Table S13. Quality Features and *P*_low_ values as input and output by seqQscorer respectively. Column prefixes designate types of quality features as follows. BowtieMI: mapping metrics from bowtie2 ran on the first fastq file for paired reads to allow comparison to single read data as used by seqQscorer. FASTQC: ordinal outputs of FastQC. TSS: percentage of counted reads in bins around transcription start sites (TSS) with relative bin position in base pairs given in full feature name. ReadsAnno: percentage of sequencing reads in different types of genomic regions described in full feature name. Accession: SRR run accession number. Dataset: GEO accession number. Plow: low-quality probability predicted by the seqQscorer tool.

Additional file 15: Review history.

## Data Availability

All the data used in this study is publicly available at the GEO database. GEO identifiers of the ChIP-seq datasets: GSE104404, GSE107734, GSE112342, GSE120738, GSE124070, GSE126571, GSE128242, GSE139377, GSE153875, GSE74230. GEO identifiers of the RNA-seq datasets: GSE100925, GSE105130, GSE108643, GSE114564, GSE116250, GSE117875, GSE119834, GSE123496, GSE125583, GSE147339, GSE148355, GSE159851, GSE25599, GSE54456, GSE73189, GSE74697, GSE76220, GSE85567, GSE101432, GSE117970, GSE133039, GSE135036, GSE136630, GSE144269, GSE147352, GSE148036, GSE151243, GSE151282, GSE155800, GSE164158, GSE164213, GSE165595, GSE173078, GSE174330, GSE179397, GSE179448, GSE182440, GSE77314, GSE79362 and GSE82177.
