# Supplementary material for: Overlooked poor-quality patient samples in sequencing data impair reproducibility of published clinically relevant datasets
Source: Genome Biol. 2024 Aug 16;25:222. doi: 10.1186/s13059-024-03331-6 (PMC11328481; doi:10.1186/s13059-024-03331-6)

Supplementary Information

Table of Contents

Quality Imbalance based on other metrics 1

Supplementary figures legends 3

Supplementary figures 5

Quality Imbalance based on other metrics

Although quality imbalance is derived using Pearson's correlation coefficients in our study, we also tested Spearman's correlation coefficients and a median-based central tendency difference 'CTDiff' as non-parametric alternatives expected to be more robust to outliers. The trends observed in the results of our study remained the same. For example, the impact of QI on the number of differential genes was the same, as shown in Fig. S4. In both cases the high QI datasets showed a much stronger slope in comparison to the low QI datasets in both paired and non-paired design experiments, meaning that gene lists are artificially inflated in high QI datasets.

The quality markers based on these metrics are also highly similar: Low-quality markers include a large number of mitochondrial and ribosomal genes and are consequently enriched in these pathways, the high-quality marker genes are enriched in pathways involving different diseases, especially cancer and those that heavily depend in a given regulator such as foxp3 or mir21 targets (see supplementary Fig. S5C and D and S6C and D). When looking at the regulators, we also observe that the low-quality markers are mainly regulated by transcription factors, while the high-quality markers seem to be regulated by miRNAs (see Fig. S5A and B and S6A and B).

In both cases the number of high-QI datasets was showing the same trend. When using Spearman's rho, the number was also 14 (35 %) with the same cut-off as with Pearson's r. When using CTDiff, which is not bound to -1 and 1 but exceeds absolute values of 1 (a correlation of 0.9 in Spearman or Pearson correlations was equivalent to a CTDiff value of greater than 11, while the majority of values remained below 1), we needed to set other cut-offs. When using 0.5 as a cut-off we observed 15 (37.5 %) high QI datasets. At a cutoff of 1, which means that the difference of the group medians exceeds the expected value of absolute inner differences, we observed 8 (20 %) high QI datasets. This more stringent selection is shown in the supplementary figures referencing CTDiff results (Fig. S4C and D and Fig. S6).

When employing a permutation significance test based on Spearman's Rho or a Wilcoxon test as a metric, with a p-value cutoff equal to 0.05, 9 (22.5 %) datasets would be considered imbalanced, a permutation test based on CTDiff leads to 10 significant datasets (25 %). However, datasets with very high QI but a low number of samples, such as GSE151282 (n = 8), would not meet the p-value cut-off of 0.05 as their small size considerably limits the test's power. Nevertheless, it is unarguable that we have a strong imbalance in the quality of the sample of this dataset, which will probably impact the analysis results, showing that aiming for measuring the effect size is favorable since many experiments will not have large sample sizes, as found in the literature. For example, if we limit the Spearman and CTDiff statistical tests only to datasets with 15 samples or more (n = 28), we still find all 9 and 10 significantly imbalanced datasets as before, resulting in 32 % and 36 %, respectively.

The choice to use Pearson's correlation coefficient to derive a quality imbalance index in our study is motivated by the fact that contrary to tested non-parametric methods, such as Spearman and CTDiff detailed above, Pearson's correlation coefficient would emphasize statistical signals from extreme observations from which we expect relevant quality-related signal and bias of expression.

Supplementary figures legends

Fig. S1. Quality Imbalance in ChIP-seq Datasets. Each panel shows a barplot depicting the quality of the samples of a given ChIP-seq dataset. Datasets with human control and disease samples were selected from the GEO database. Bars are proportional to the low-quality probability of a sample calculated by the machine-learning tool seqQscorer. The QI calculated between the low-quality probabilities and the numerical sample group codes (0 for control and 1 for disease) is shown together with the p-value of the corresponding correlation test.

Fig. S2. Impact of Quality Imbalance (QI) on the Number of Differential Genes. Representation is as in Figure 3, but here we compare results for datasets not using paired samples (panels A and B) to datasets using paired samples (panels C and D).

Fig. S3. Quality Markers and Known Disease Genes. For 20 diseases associated with the 40 studied datasets and with more than 300 known genes, boxplots represent disease genes that were derived from the literature and selected by a false discovery rate < 0.05 (Y axis). The lower the discovery rate, the stronger the association of a gene with a disease in the literature. Boxes represent the distribution of disease genes that are also high-quality markers (negative), disease genes that are also low-quality markers (positive), and disease genes that are neither high- nor low-quality markers (none).

Fig. S4. Impact of Quality Imbalance (QI) on the Number of Differential Genes, when employing other base metrics. The figure shows distribution of differential genes versus number of samples over high vs low QI datasets, when employing Spearman's correlation coefficient or CTDiff to define QI.

Fig. S5. Gene set enrichment analysis. Analogous to Figure 5 the pathway and regulation enrichment analysis of the low-quality markers identified on the basis of Spearman's Rho based QI.

Fig. S6. Gene set enrichment analysis. Analogous to Figure 5 the pathway and regulation enrichment analysis of the low-quality markers identified on the basis of CTDiff based QI.

Fig. S7. Quality Features used by seqQscorer in comparison to P_{low}. A The panel shows from left to right: A heatmap of P_{low} (white to dark orange with increasing P_{low}), FastQC's ordinal output (red: Fail or 0, yellow: Warn or 1, green: Pass or 2), and bowtie2's mapping metrics (0 to 100 in percent of reads, blue to red, respectively) used with seqQscorer's mixed mapping strategy (BowtieMI). **B** Distribution of uniquely mapped reads statistics of the samples over all datasets and colored with 4 colors representing the quartiles of P_{low} values associated with the samples. The worse the quality (high P_{low}) the lower the uniquely mapped reads. **C** Distribution of Sequence duplication level within the sample's reads as returned by FastQC: Fails (0) and Warns (1) are more abundant in samples with high P_{low}. Quartiles are colored as in B.

Fig. S8. Distribution of samples over P_{low} in 9 example datasets. Examples of datasets ranging from the lowest to the highest QI of all observed datasets. The two datasets with the highest QI, GSE164213 and GSE135036 respectively, show the disease group as the lower- and higher-quality fraction, respectively. There seem to be no systematic preference for biological groups to be in either fraction.

Supplementary figures

Fig S1 - Quality Imbalance in ChIP-seq Datasets.

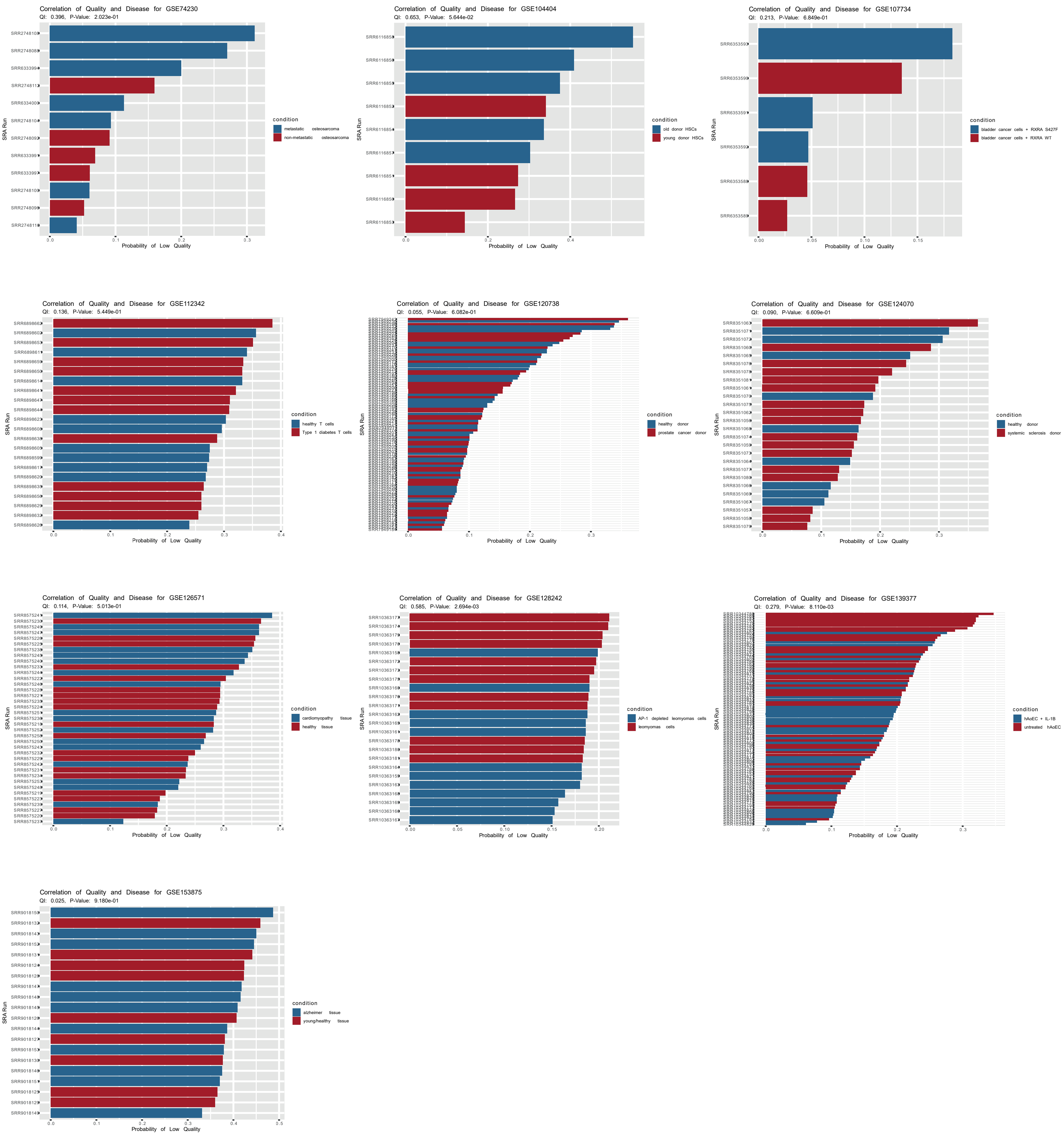

Fig S2.
Impact of Quality Imbalance (QI) on the Number of Differential Genes.

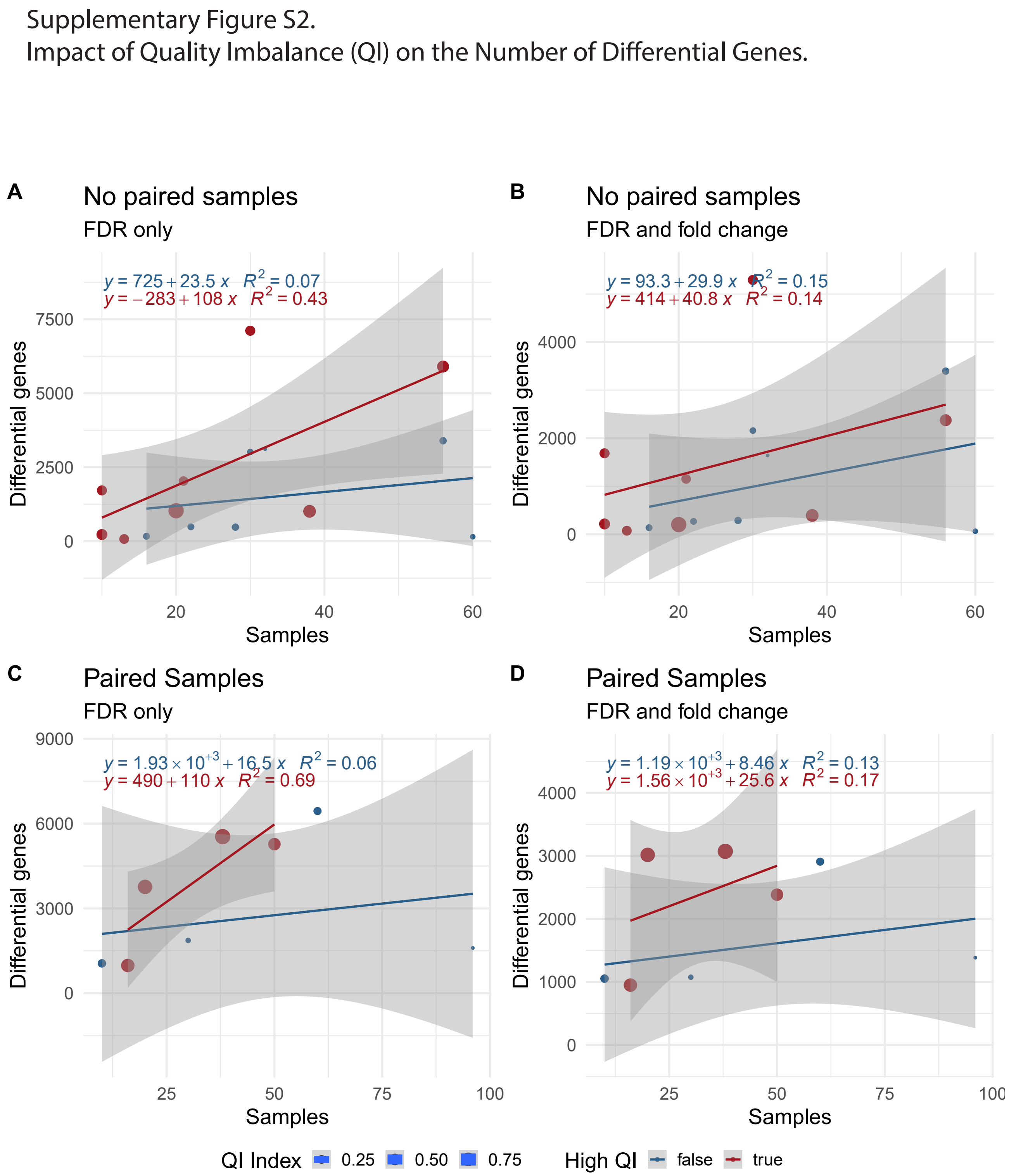

Fig S3.
Quality Markers and Known Disease Genes

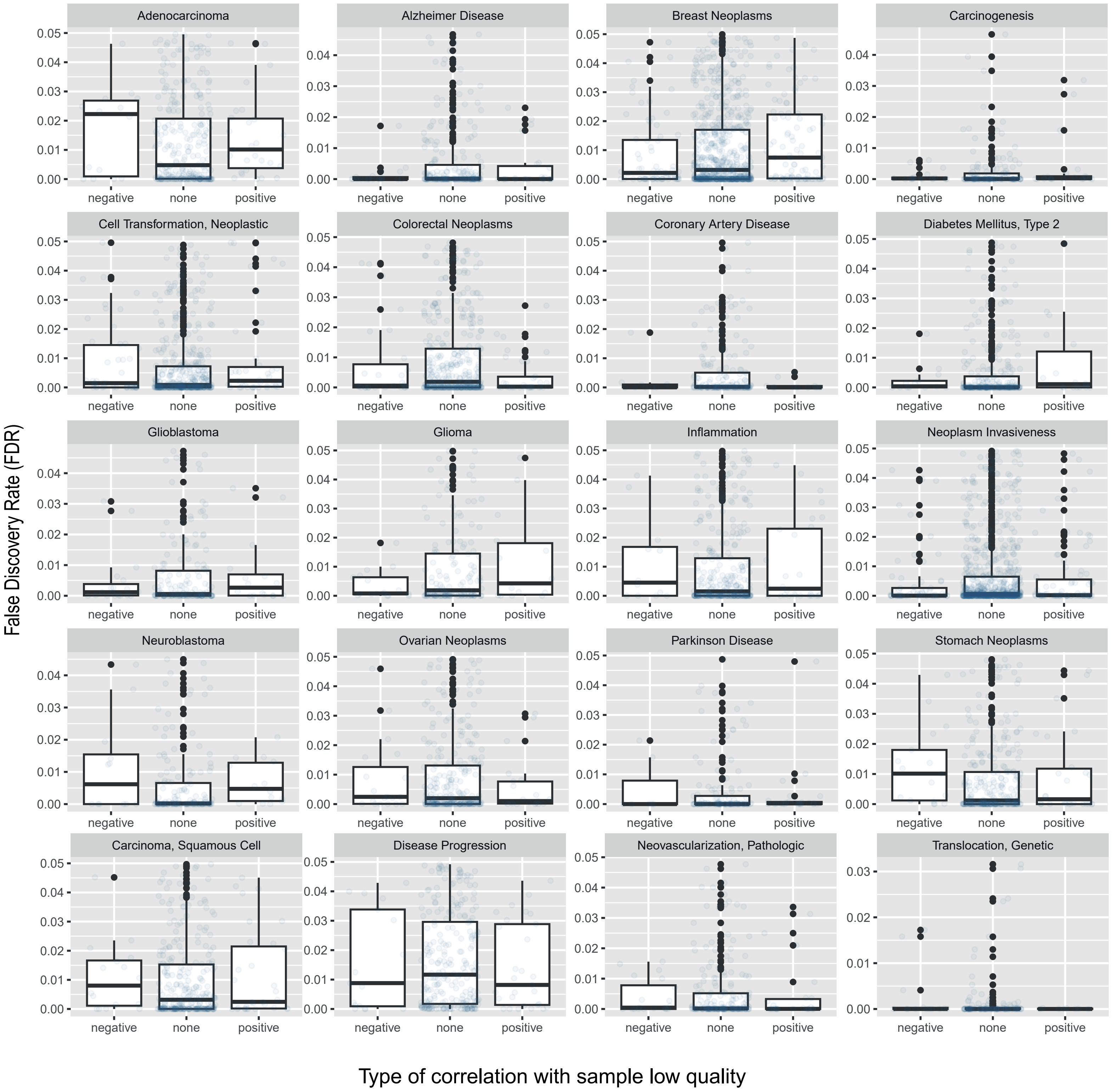

Fig S4.
Impact of Quality Imbalance (QI) on the Number of Differential Genes, when employing other base metrics.

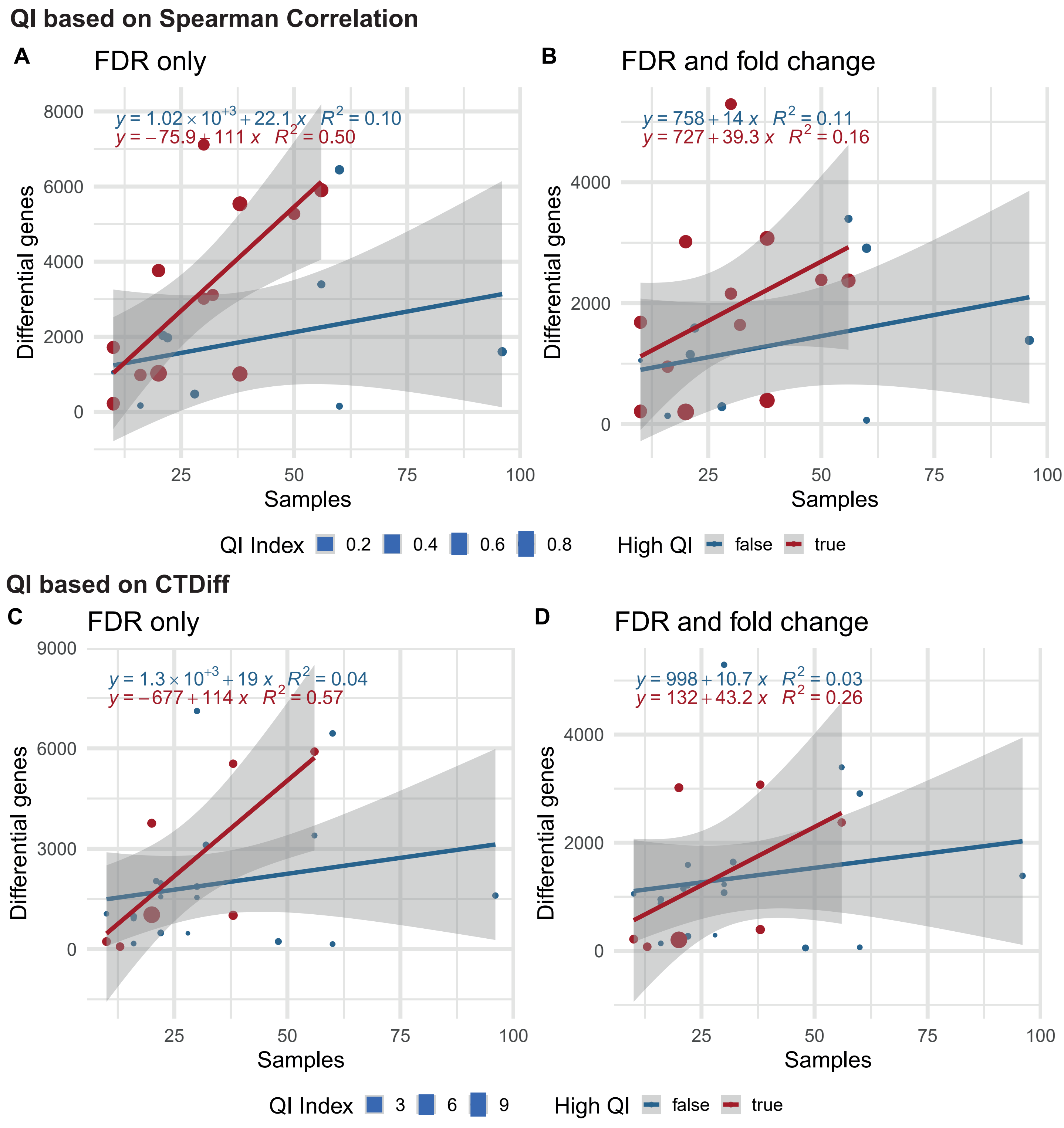

Fig S5.
Gene set enrichment analysis - Spearman's Rho

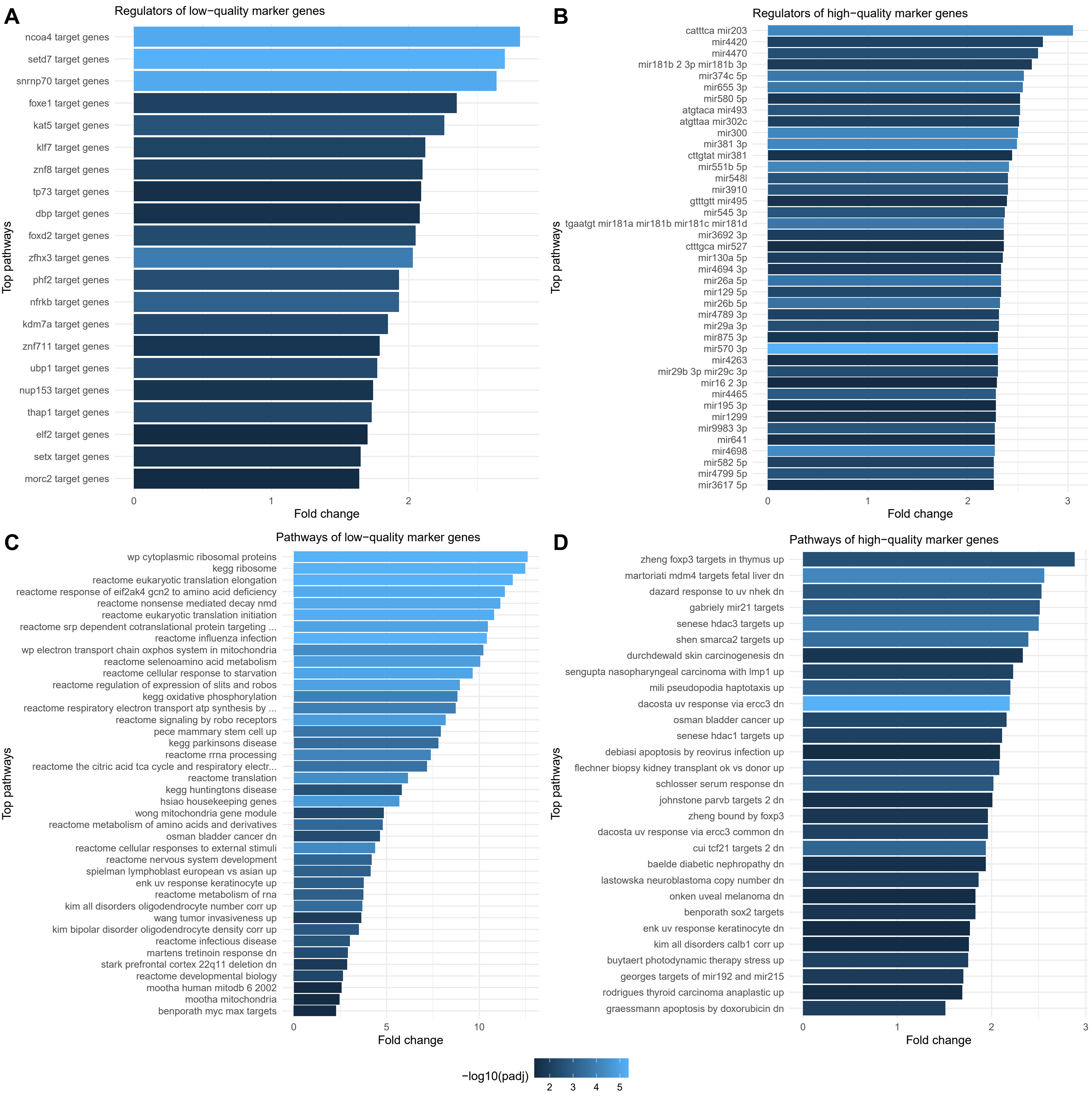

Fig S6.
Gene set enrichment analysis - CTDiff

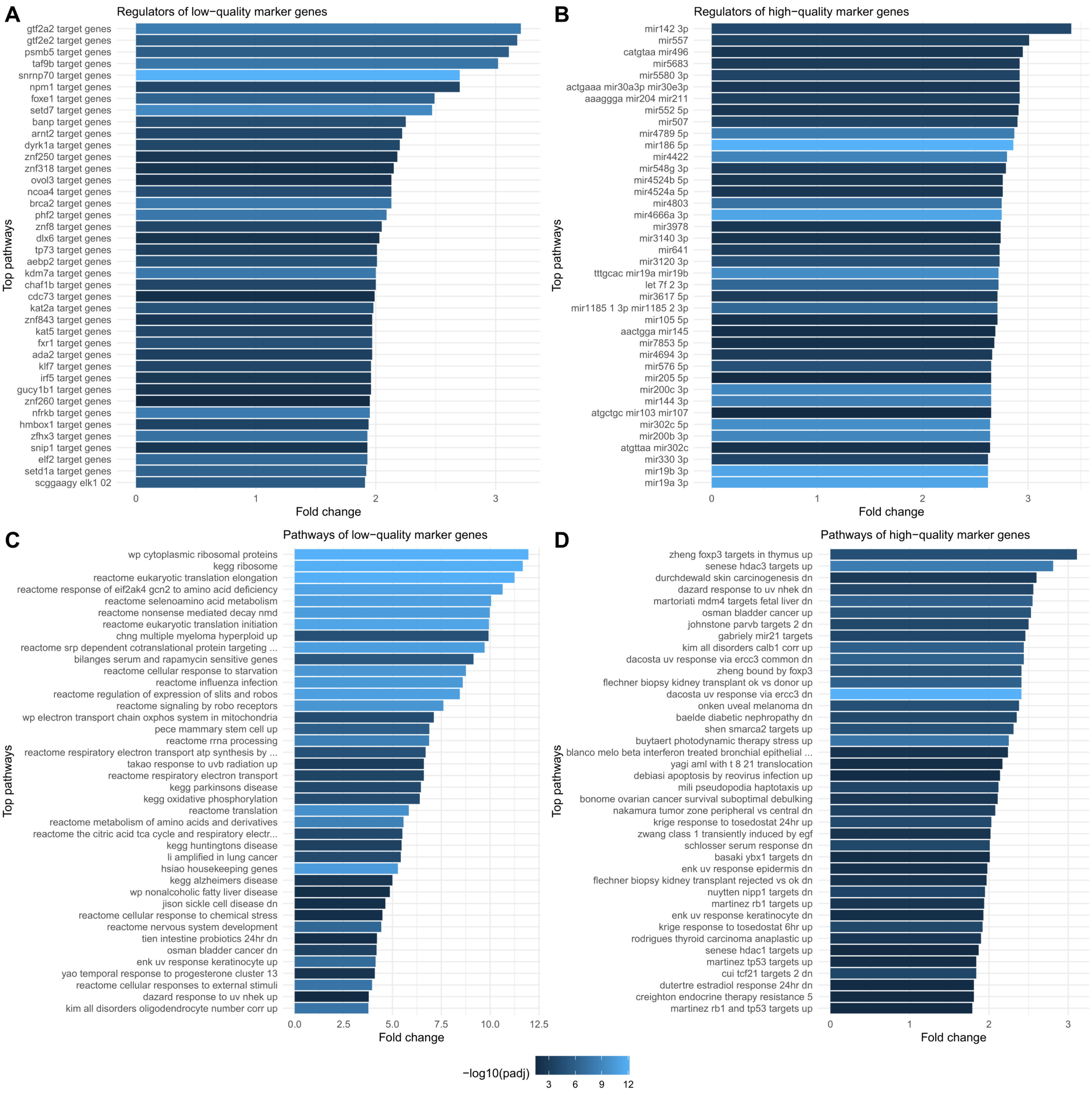

Fig S7.
Quality Features used by seqQscorer in comparison to Plow.

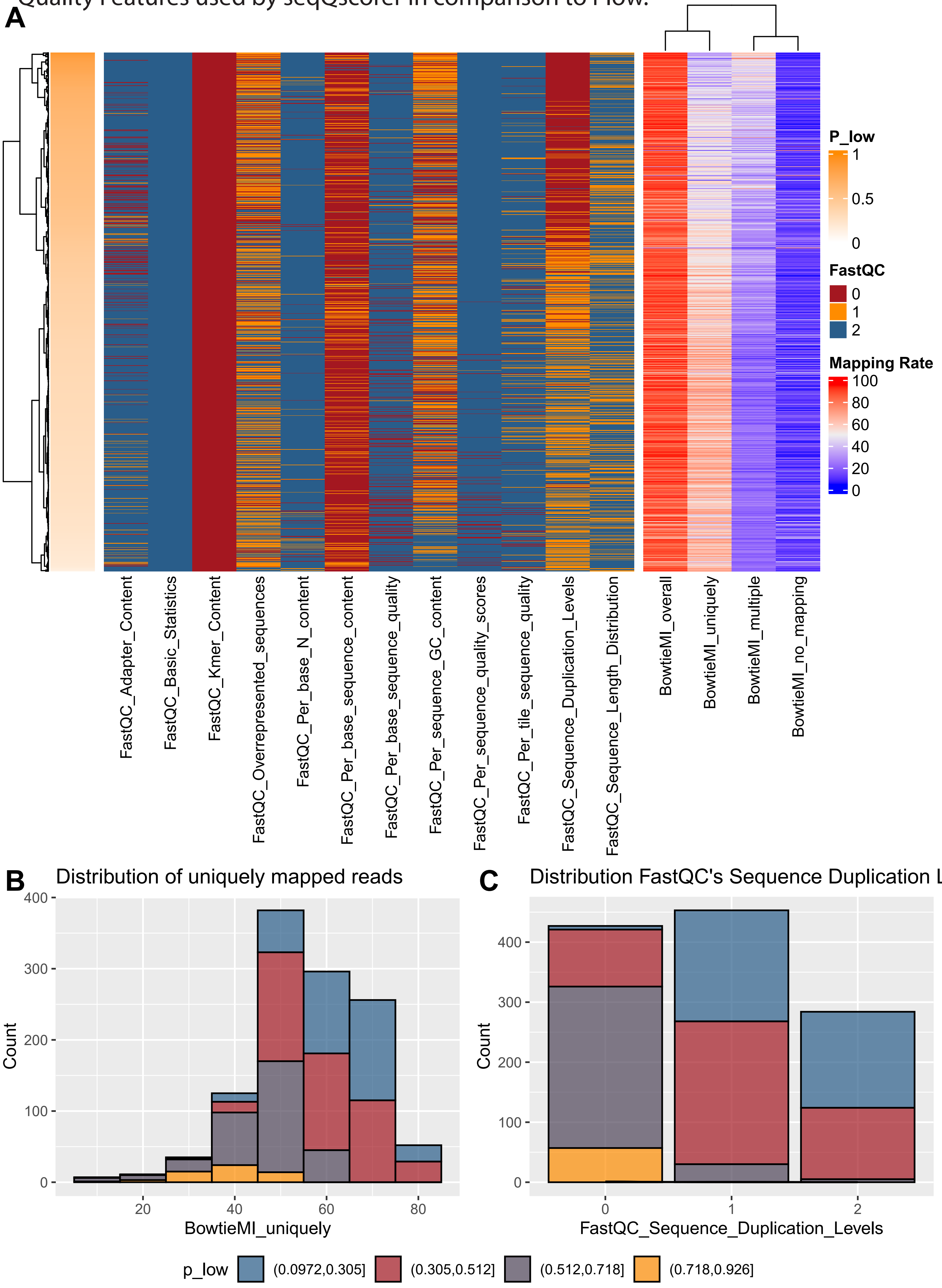

Fig S8.
Distribution of samples over Plow in 9 example datasets

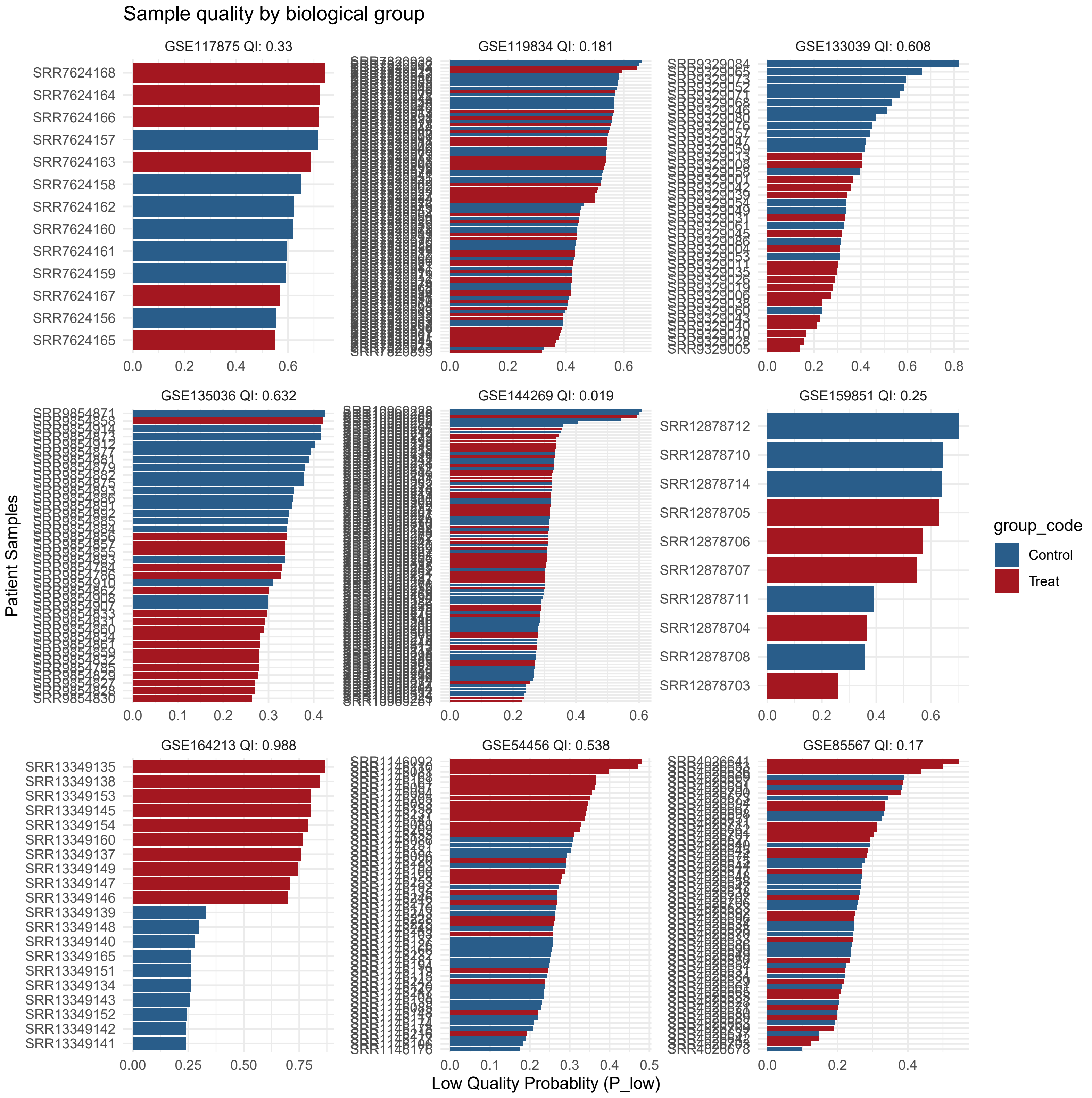

Supplement: Supplementary file 1 — Additional file 1. Supplementary information and figures. This file contains a section named “Quality Imbalance based on other metrics” and the supplementary figures (Fig. S1 to Fig. S8). [file 13059_2024_3331_MOESM1_ESM.pdf]
