# Supplementary material for: Overlooked poor-quality patient samples in sequencing data impair reproducibility of published clinically relevant datasets
Source: Genome Biol. 2024 Aug 16;25:222. doi: 10.1186/s13059-024-03331-6 (PMC11328481; doi:10.1186/s13059-024-03331-6)
Supplement: Supplementary file 15 — Additional file 15: Review history. [file 13059_2024_3331_MOESM15_ESM.docx]

Review History

**First round of review**

**Reviewer 1**

**Were you able to assess all statistics in the manuscript, including the appropriateness of statistical tests used?**

Yes, and I have assessed the statistics in my report.

**Comments to author:**

Sprang et al, test whether differences in data quality between samples of the same RNASeq (and ChIPSeq) dataset bias the analysis of differential expression using a set of 40 previously published RNA-seq datasets. Quality assessment is calculated using a tool called seqQscorer published by the same authors in 2021 that uses machine learning and various basic metrics of data quality to calculate the probability that an omics sample is of low quality. The idea is that imbalance in data quality between control and disease samples is an omitted variable that confounds analyses causing false positives when calling differentially expressed genes and differential ChIP binding. They show that high quality imbalance between control and disease samples is associated with a higher number of differentially expressed genes and that these genes are enriched for specific functions/targets of certain regulators.

The idea behind the paper is interesting. Although it is expected that imbalance in quality between control and test samples can introduce artefacts, in my opinion, it is worth proving the point and investigating what kind of gene signature this bias generates. Nevertheless, in my opinion, from what is shown in Figures 2 and 3, the effect of quality imbalance on the # of differentially expressed genes is not very strong and also the genes up/down regulated are not very consistent between datasets. My conclusion from this work is that QI should be avoided because it introduces noise but if it cannot be avoided (I suspect usually the case), then it is not easy to account for.

Major comments

Figure 2: I understand that the four points per dataset shown in this figure correspond to the four artificial subsets generated so as to be two of high quality imbalance and two of low quality imbalance. Looking at the values on the x axis of the four samples per dataset, this hasn't worked as intended. In the best case (GSE144269 and GSE74697) there is a continuum of QI from low to high rather than two low and two high and in the worst case (GSE105130) there is one sample of low QI and 3 of high QI. The regression on these four points is questionable in my opinion.

I presume that disease samples are more likely to be low quality than high quality compared to control samples. Therefore real disease genes could be overrepresented in quality markers. The authors comment on this at the end of the results section and in the discussion. This is concerning and makes the identification of quality marker genes not particularly useful.

Minor comments

Line 187: "indicating that this problem exists in other assays too". The authors define quality imbalance using arbitrary thresholds and up to this line, the reader is simply presented with the number of datasets that pass this threshold. In principle there is no evidence that a quality imbalance index of >=0.3 is a problem. I suggest to replace "this problem" with "quality imbalance".

Figure 2: The grey area of the figure is not explained in the figure legend.

Figure 2 vs Figure 3: This is a minor point but it is not elegant that Figure 2 and Figure 3 that aim to show the same thing (QI vs # of diff genes) are plotted differently. Figure 3 shows QI as circle size while putting # of samples on the x-axis. It would make more sense to plot QI on x and # of diff genes on y in both figures and add # of samples as circle size. Alternatively, the authors could add the 4 points of the artificially generated datasets using different types of points in figure 3, instead of plotting them in Figure 2. They would appear as 2 hi QI points and 2 low QI points (per dataset) at x-axis value 10 of Figure 3. This is just a recommendation to help readability.

Some references are not correctly formatted.

Table S1: for sample GSE164158 the authors have a note that this is a "chinese study". I don't understand what this comment is about. Are the authors chinese (if so, no relevance) or are the samples from chinese individuals? What about all the rest of the samples?

**Reviewer 2**

**Were you able to assess all statistics in the manuscript, including the appropriateness of statistical tests used?**

Yes, and I have assessed the statistics in my report.

**Comments to author:**

The manuscript systematically analyzed the impact of data quality of clinically-relevant deep sequencing data, including RNA-seq and histone modification ChIP-seq data, on the detection of differentially expressed genes (DEGs) and differentially enriched regions from these data. Consistent with many previous reports, they showed that these datasets often showed obvious quality imbalance (QI) between disease and control samples. Importantly, they further found that, when the sample size was similar, a higher number of DEGs were identified from the datasets showing greater QI. They further identified low- or high-quality marker genes whose expression level showed a significant correlation with the sample quality, and found they tend to be identified as quality marker genes in multiple datasets and are enriched in various biological pathways, including mitochondria and ribosomal pathways as well as some disease related pathways, such as neuro-degenerative and cancer diseases.

In general, this work can help researchers to improve the design of data analysis in many clinically-relevant studies using RNA-seq and ChIP-seq techniques as well as the interpretation of the results. However, there are still several major concerns that need to be well addressed:

1. In the current manuscript, the evaluation of sample quality simply relied on the P_low statistic reported by the seqQscorer tool. The authors should provide a number of intuitive metrics about the quality of individual samples in different sample groups to give a systematic description about why some of them were labeled as low-quality ones.

2. The authors defined a quality imbalance (QI) index, which was calculated as "the Pearson's correlation coefficient (PCC) between P_low values and the sample group numerical codes (0 for control and 1 for disease)", to quantitatively describe the level of quality imbalance for each dataset and then identify the high-QI and low-QI datasets by using the absolute value of QI index >0.30 or <0.18 as the cutoff. However, by a simple transformation of the formula, it can be easily seen that the PCC between a continuous variable and a binary variable actually describes the difference between the mean of the continuous variable in the two groups of observations determined by the value of the binary variable (e.g., disease versus control). Thus, a more rigorous statistical test should be used to determine whether a significant QI was observed for each dataset.

3. The authors detected thousands of low-quality marker genes whose expression significantly correlates with the P_low in more than one data sets, which account for a substantial proportion of the differential genes between disease versus control samples detected from the relevant datasets. Moreover, these genes were significantly enriched in many biological pathways including some disease-related ones. Actually, some of these disease-related pathways frequently showed up in the functional enrichment analysis of the differential genes detected from bulk RNA-seq data in many previous studies. Could the authors design a statistical method or practical strategy to remove or reduce the impact of the quality marker genes/regions in differential RNA/ChIP-seq analysis? If possible, also their impact on subsequent functional enrichment analysis? A few recently published tools for differential RNA-seq or ChIP-seq analysis claimed that they have considered the scenarios that the sample groups for differential analysis may be associated with different levels of heterogeneity or data quality. Not sure whether their strategies may be incorporated or borrowed to have an improved differential analysis between sample groups with distinct quality indexes.

Some minor concerns:

1. The individual subfigures of figure 1 were not explicitly quoted and described in the Results sections in the current manuscript.

**Authors Response**

**Point-by-point responses to the reviewers’ comments:**

Reviewer #1

Major comments

- Figure 2: I understand that the four points per dataset shown in this figure correspond to the four artificial subsets generated so as to be two of high quality imbalance and two of low quality imbalance. Looking at the values on the x axis of the four samples per dataset, this hasn't worked as intended. In the best case (GSE144269 and GSE74697) there is a continuum of QI from low to high rather than two low and two high and in the worst case (GSE105130) there is one sample of low QI and 3 of high QI. The regression on these four points is questionable in my opinion.

*ANSWER => We agree, that a regression of just four points is questionable and we only meant to help the reader observing the trends. Nevertheless, this point as well as this reviewer’s note to the non-ideal distribution of points are valid and led us to improve this simulation overall. We built bigger data subsets to reduce the impact of chance on the DEG, as in small groups the DEGs can vary more strongly. Instead of just taking the 10 samples of lowest and highest quality of a given dataset and biological group, we took 20 samples per subsets. We also increased the number of subsets per dataset to fit the regressions with more confidence from 4 to 12 data subsets (12 points to compute the regression). To allow this new analysis, we used the following 3 source datasets because they have a minimum of 50 samples and a broad distribution of Plow values among their samples: GSE105130, GSE174330 and GSE54456. We added new explanations of the changed procedure in line 111-124, 239, 361-375, 420-427.*

- I presume that disease samples are more likely to be low quality than high quality compared to control samples. Therefore real disease genes could be overrepresented in quality markers. The authors comment on this at the end of the results section and in the discussion. This is concerning and makes the identification of quality marker genes not particularly useful.

*ANSWER => We agree with the reviewer that it is an important hypothesis to consider. Yet, we did not observe it as a systematic pattern in our ChIP-seq and RNA-seq selection of datasets (Figure S1 and S5, respectively) but it may still be true for some diseases. Nevertheless, spinning this thought further, we could hypothesize that the opposite also can be true and that some disease genes, even if they have been shown repeatedly in the literature, may be more related to quality artifacts than to the actual underlying disease, or at least that their importance as a disease maker gene can be overplayed by a confounding quality artifacts. We discussed these points more clearly in the discussion (line 251 and 278-281). In this context we’d like to emphasize again, that the potential quality marker genes in this work were derived from the least imbalanced datasets. Even in datasets showing high concurrence of quality between the biological groups we get a clear signal, that fits to the expectation of finding mitochondrial and ribosomal genes and pathways and should be less biased by the group QI (See Line 136-157 and 251).*

Minor comments

- Line 187: "indicating that this problem exists in other assays too". The authors define quality imbalance using arbitrary thresholds and up to this line, the reader is simply presented with the number of datasets that pass this threshold. In principle there is no evidence that a quality imbalance index of >=0.3 is a problem. I suggest to replace "this problem" with "quality imbalance".

*ANSWER => We agree and have changed the text accordingly. (Line 108)*

- Figure 2: The grey area of the figure is not explained in the figure legend.

*ANSWER => We addressed this point, see line 428.*

- Figure 2 vs Figure 3: This is a minor point but it is not elegant that Figure 2 and Figure 3 that aim to show the same thing (QI vs # of diff genes) are plotted differently. Figure 3 shows QI as circle size while putting # of samples on the x-axis. It would make more sense to plot QI on x and # of diff genes on y in both figures and add # of samples as circle size. Alternatively, the authors could add the 4 points of the artificially generated datasets using different types of points in figure 3, instead of plotting them in Figure 2. They would appear as 2 hi QI points and 2 low QI points (per dataset) at x-axis value 10 of Figure 3. This is just a recommendation to help readability.

*ANSWER => While we acknowledge that the reviewer has a point, our intention was that the difference between the plots would emphasize the QI in Figure 2, as the sizes of the subsetted datasets are all equal (10 samples before, now 20) and thus there is no impact of sample size on DEGs. In Figure 3, the sample size was chosen as the x-axis variable to show the natural increase in differential genes with dataset size, as also discussed in results and discussion, while the QIs impact is indicated by the color to emphasize the different QI groups and show that the effect persists even in the presence of other strong effects on the number of DEGs. The point size was additionally introduced to give an impression of the state of the datasets, that are close in QI to the other respective group of samples concerning their QI.*

- Some references are not correctly formatted.

*ANSWER => We added the link to seqTK’s citation, which was missing. The picard toolkit does not have an author’s name, but this is in accordance with their citation statement in the github repo, we also added the link. We will of course provide additional information about the references to the editor later, should any more be required.*

- Table S1: for sample GSE164158 the authors have a note that this is a "chinese study". I don't understand what this comment is about. Are the authors chinese (if so, no relevance) or are the samples from chinese individuals? What about all the rest of the samples?

*ANSWER => All notes in the table referred to by this reviewer reflect the observable homogeneity of the studies used. The more homogeneous, the better for this study, as then fewer confounding factors are to be expected. We considered ethnicity and nationality as probable indicators of homogeneity, which is why we also incorporated such information if it is clearly indicated that the sample set is only of a certain ethnicity/nationality. For example, there are also “Study from South Korea” (GSE148355) as well as "All subjects were European Caucasian" (GSE54456) and various information about the distribution of sex in between the groups. We rephrased these notes to be more clear about their intended nature.*

Reviewer #2:

- 1. In the current manuscript, the evaluation of sample quality simply relied on the P_low statistic reported by the seqQscorer tool. The authors should provide a number of intuitive metrics about the quality of individual samples in different sample groups to give a systematic description about why some of them were labeled as low-quality ones.

*ANSWER => seqQscorer uses metrics from the reads mapping (Bowtie2) as well as quality software outputs (fastQC) and percentages of reads in genomic regions and bins around the TSS. So, the P_low statistic is based on the same information a human user would also observe, as we showed in our work “Statistical guidelines for quality control of next-generation sequencing techniques” (DOI: 10.26508/lsa.202101113). We added some plots showcasing the distribution of samples over all datasets especially over the “uniquely mapped reads” as well as FastQC’s “Sequence Duplication Levels” (Supplementary Figure S4) and added a table containing this information, as well as the less human interpretable counts of reads in genomic regions and bins around the TSS (Supplementary Table S12). They are referenced in Lines 329-336, 477-485, 522-529. However, it needs to be added here, that the study at hand does not use a cut-off of any kind directly on those metrics to classify a given sample as low- or high- quality, but only utilizes P_low from seqQscorer as a score, specifically to compute the QI index. This was done deliberately, to be able to catch more subtle differences in quality, even when the imbalance between groups does not lead to a strong loss in quality in a given sample population.*

- 2. The authors defined a quality imbalance (QI) index, which was calculated as "the Pearson's correlation coefficient (PCC) between P_low values and the sample group numerical codes (0 for control and 1 for disease)", to quantitatively describe the level of quality imbalance for each dataset and then identify the high-QI and low-QI datasets by using the absolute value of QI index >0.30 or <0.18 as the cutoff. However, by a simple transformation of the formula, it can be easily seen that the PCC between a continuous variable and a binary variable actually describes the difference between the mean of the continuous variable in the two groups of observations determined by the value of the binary variable (e.g., disease versus control). Thus, a more rigorous statistical test should be used to determine whether a significant QI was observed for each dataset.

*ANSWER => We agree that the QI is not a test of significance for differences between groups in terms of low-quality probability, but this is by design: While a test such as the Kruskal-Wallis-test could be applied, it loses statistical power for small sample sizes, which are common in biological settings and also sometimes result from the stratification of the samples to ensure maximum homogeneity in our study. Significance can also be expected from such a test, if the difference is small, but the groups respective variance is also very small or if the sample size is big. Therefore, we opted to measure an effect size having a lower dependency to sample size rather than the significance of a test. By assigning a binary numerical value to the groups and run a PCC, the PCC is equivalent to a Point-biserial correlation, which is a standard way of comparing linear relationships of dichotomous variables to continuous variables. We added these points to the manuscript in lines 338-339. To give the reader a more visual feeling of how the QI is measured we added a new supplementary figure, showing the distribution of samples over P_low by group with Supplementary figure S5 (Lines 486-490). Nevertheless, the Kruskal-Wallis-test shows a clear trend to significant p-values for the high QI datasets (but also for some of the low-QI datasets, due to their size and relative homogeneity of groups). It should be noted, that when only the larger datasets are considered (number of samples > 15), for which the statistical power should not be too low, the proportion of significant datasets is similar to the number of datasets passing our QI threshold: 10 (~36%) out of 28 datasets.*

- 3. The authors detected thousands of low-quality marker genes whose expression significantly correlates with the P_low in more than one data sets, which account for a substantial proportion of the differential genes between disease versus control samples detected from the relevant datasets. Moreover, these genes were significantly enriched in many biological pathways including some disease-related ones. Actually, some of these disease-related pathways frequently showed up in the functional enrichment analysis of the differential genes detected from bulk RNA-seq data in many previous studies. Could the authors design a statistical method or practical strategy to remove or reduce the impact of the quality marker genes/regions in differential RNA/ChIP-seq analysis? If possible, also their impact on subsequent functional enrichment analysis? A few recently published tools for differential RNA-seq or ChIP-seq analysis claimed that they have considered the scenarios that the sample groups for differential analysis may be associated with different levels of heterogeneity or data quality. Not sure whether their strategies may be incorporated or borrowed to have an improved differential analysis between sample groups with distinct quality indexes.

*ANSWER => A practical strategy is summarized in the following sentence of the discussion (L272-274): “Provided that pairs of samples are not confounded with quality differences or batch processing, designing datasets with sample pairing and applying appropriate statistics (paired statistical tests) may be the optimal way to maximize relevance and reproducibility of RNA-seq results“. In previous work, dealing with batch effects, we showed, that P_low can be used as a covariate to correct for some batch effects, however, the stronger these artifacts are confounded with the group the harder they are to detect and correct (DOI: 10.1186/s12859-022-04775-y). For example, state of the art artifact removal software (sva, RUVseq), uses the group information as prior information, which will ultimately lead to these highly confounded artifacts going undetected, especially those we observed in this work. The only way to really know that there are expression patterns confounded with the quality in a given dataset is to replicate our workflow here and run seqQscorer, obtain P_low and then look for correlations of gene expression with P_low in the samples of the respective groups of the dataset. Another strategy to account for sample quality would be to use P_low as a covariate within DESeq2s differential analysis. However, by using published datasets such as in our study, there is not an easy way of verifying that we would actually be improving the results, as depending on context, even simple metrics such as the number of enriched pathways are not straightforward to interpret. For example, in large datasets we may see a decrease, as we could reduce false positives but for smaller sets an increase may be desirable. While we expect quality artifacts to mostly introduce false positives, it cannot be excluded that they may act in the other direction and dampen the differences between two actually differentially expressed transcripts, thereby inducing false negatives. To actually measure the impact of such statistical strategies to control the quality-related genes in significant analysis results, we would need to design an experiment from the ground up with the aim to actively inject errors in the wet-lab protocol and see how these changes impact the quality as well as the expression of genes. While this is out of scope for this work, we plan to partner up with wet-labs in the future to follow up in this direction.*

Some minor concerns:

- 1. The individual subfigures of figure 1 were not explicitly quoted and described in the Results sections in the current manuscript.

*ANSWER => We added the references and some description. (Line 100 – 108)*

**Second round of review**

**Reviewer 1**

The authors have addressed my criticisms.

**Reviewer 2**

The revised manuscript has addressed some of my previous concerns, but the others largely remain there.

In regard to the second of my previous comments, I do not mean to simply replace the PCC cutoff by cutoff of statistical significance derived from a suitable test. As we know, PCC values can be dominated by a small number of outlier observations. So, it’s better to also take this into consideration when quantifying the QI between different sample groups.

For the third of my previous comments, I think it’s highly necessary to design a practical statistical method or workflow to reduce the impact of the quality marker genes/regions in differential RNA/ChIP-seq analysis or, at least, their impact on subsequent functional enrichment analysis. As an example, the neural disease pathways (kegg huntingtons disease, kegg alzheimers disease, kegg parkinsons disease) enriched by the low-quality marker genes (showed in Figure 5C) frequently showed up in the list of pathways enriched by the differentially expressed genes identified in many previous studies that have no obvious relevance with neural system development or diseases. Typically, readers of this paper may consider the low-quality markers as blacklisted genes/peaks and would prefer to simply remove them from their analyses, which, definitely, is not the desired way. After all, the identification of these genes/genomic regions as quality markers does not mean they have no functional relevance.

**Authors Response**

**Point-by-point responses to the reviewers’ comments:**

Reviewer #2:

The revised manuscript has addressed some of my previous concerns, but the others largely remain there.

- In regard to the second of my previous comments, I do not mean to simply replace the PCC cutoff by cutoff of statistical significance derived from a suitable test. As we know, PCC values can be dominated by a small number of outlier observations. So, it’s better to also take this into consideration when quantifying the QI between different sample groups.

*Answer:*

*We tested other correlation and effect size measures and associated permutation tests such as Spearman’s Rho and CTDiff (central tendency difference; Lotsch and Ultsch, PLoS One 2020). Both are non-parametric statistics more robust to outliers than Pearson’s R, as they rely on ranking and medians, respectively. They led to similar numbers of high QI datasets, and the results when running the downstream analysis for low- and high-quality markers are highly similar to our former version. Nevertheless, we settled for staying with Pearson’s R in the main manuscript. We argue that its potential weakness in being biased towards quality outliers is also its strength in our case, because we are studying the impact of quality differences that are especially driven by outliers which can impact downstream analysis, such as differential expression and enrichment analyses.*

*The new experiments and results are in the new Supplementary information 1 file and in lines 249-251, 386-396, and 527-535 (new Supplementary Figures S4, S5, S6).*

Reviewer #2:

For the third of my previous comments, I think it’s highly necessary to design a practical statistical method or workflow to reduce the impact of the quality marker genes/regions in differential RNA/ChIP-seq analysis or, at least, their impact on subsequent functional enrichment analysis. As an example, the neural disease pathways (kegg huntingtons disease, kegg alzheimers disease, kegg parkinsons disease) enriched by the low-quality marker genes (showed in Figure 5C) frequently showed up in the list of pathways enriched by the differentially expressed genes identified in many previous studies that have no obvious relevance with neural system development or diseases. Typically, readers of this paper may consider the low-quality markers as blacklisted genes/peaks and would prefer to simply remove them from their analyses, which, definitely, is not the desired way. After all, the identification of these genes/genomic regions as quality markers does not mean they have no functional relevance.

*Answer:*

*We fully agree with the reviewer that the genes should not be just removed from the analysis and that while we hypothesize that there may be a systemic bias that extends the datasets we observe here, these genes still can have functional relevance. Indeed, in lines 307 to 313, we hypothesize that the genes appear as quality markers because of their role in the regulation of the cell equilibrium.*

*We experimented with using the quality information gained in our workflow to mitigate the impact of the quality bias on the pathway analysis by checking the number of pathways enriched in low-quality marker genes in all datasets and observing how this percentage changes when introducing the low-quality probability P_low as a covariate in the differential expression analysis (DESeq2 package), as well as when using it to remove outliers in regard to sample quality from the datasets. While the impact of using P_low as a covariate was rather small, removing quality-outlier samples from the analysis worked remarkably well.*

*These new results are included now in lines 31f, 194-204 (new supplementary Table S12), 316-319 and 436-450.*

**Third round of review**

**Reviewer 2**

The authors have satisfactorily addressed my previous concerns.
